# Retinal Proteome Profiling of Inherited Retinal Degeneration Across Three Different Mouse Models Suggests Common Drug Targets in Retinitis Pigmentosa

**DOI:** 10.1016/j.mcpro.2024.100855

**Published:** 2024-10-09

**Authors:** Ahmed B. Montaser, Fangyuan Gao, Danielle Peters, Katri Vainionpää, Ning Zhibin, Dorota Skowronska-Krawczyk, Daniel Figeys, Krzysztof Palczewski, Henri Leinonen

**Affiliations:** 1 School of Pharmacy, Faculty of Health Sciences, University of Eastern Finland, Kuopio, Finland; 2 Center for Translational Vision Research, Department of Ophthalmology, Gavin Herbert Eye Institute, University of California, Irvine, Irvine, California, USA; 3 Department of Physiology and Biophysics, University of California, Irvine, California, USA; 4 Ottawa Institute of Systems Biology, University of Ottawa, Ottawa, Ontario, Canada; 5 Department of Biochemistry, Microbiology and Immunology, University of Ottawa, Ottawa, Ontario, Canada; 6 Department of Chemistry, University of California, Irvine, California, USA; 7 Department of Molecular Biology and Biochemistry, University of California, Irvine, California, USA

**Keywords:** inherited retinal degenerations, retinitis pigmentosa, retinal proteome, retinal degeneration, rd10, P23H, *Rpe65*^−/−^

## Abstract

Inherited retinal degenerations (IRDs) are a leading cause of blindness among the population of young people in the developed world. Approximately half of IRDs initially manifest as gradual loss of night vision and visual fields, characteristic of retinitis pigmentosa (RP). Due to challenges in genetic testing, and the large heterogeneity of mutations underlying RP, targeted gene therapies are an impractical largescale solution in the foreseeable future. For this reason, identifying key pathophysiological pathways in IRDs that could be targets for mutation-agnostic and disease-modifying therapies (DMTs) is warranted. In this study, we investigated the retinal proteome of three distinct IRD mouse models, in comparison to sex- and age-matched wild-type mice. Specifically, we used the Pde6β^Rd10^ (rd10) and Rho^P23H/WT^ (P23H) mouse models of autosomal recessive and autosomal dominant RP, respectively, as well as the *Rpe65*^−/−^ mouse model of Leber’s congenital amaurosis type 2 (LCA2). The mice were housed at two distinct institutions and analyzed using LC-MS in three separate facilities/instruments following data-dependent and data-independent acquisition modes. This cross-institutional and multi-methodological approach signifies the reliability and reproducibility of the results. The large-scale profiling of the retinal proteome, coupled with *in vivo* electroretinography recordings, provided us with a reliable basis for comparing the disease phenotypes and severity. Despite evident inflammation, cellular stress, and downscaled phototransduction observed consistently across all three models, the underlying pathologies of RP and LCA2 displayed many differences, sharing only four general KEGG pathways. The opposite is true for the two RP models in which we identify remarkable convergence in proteomic phenotype even though the mechanism of primary rod death in rd10 and P23H mice is different. Our data highlights the cAMP and cGMP second-messenger signaling pathways as potential targets for therapeutic intervention. The proteomic data is curated and made publicly available, facilitating the discovery of universal therapeutic targets for RP.

## Highlights

• Proteomic analyses reveal retinal proteome data from three mouse models of degeneration.
• Retinitis pigmentosa and Leber congenital amaurosis type 2 models exhibit distinct proteomic changes.
• Rd10 and P23H retinitis pigmentosa models display remarkable convergence in proteomic phenotypes.
• Mutation-agnostic therapies are feasible due to shared pathological phenomena in retinitis pigmentosa.

## Graphical Abstract

**Figure:**
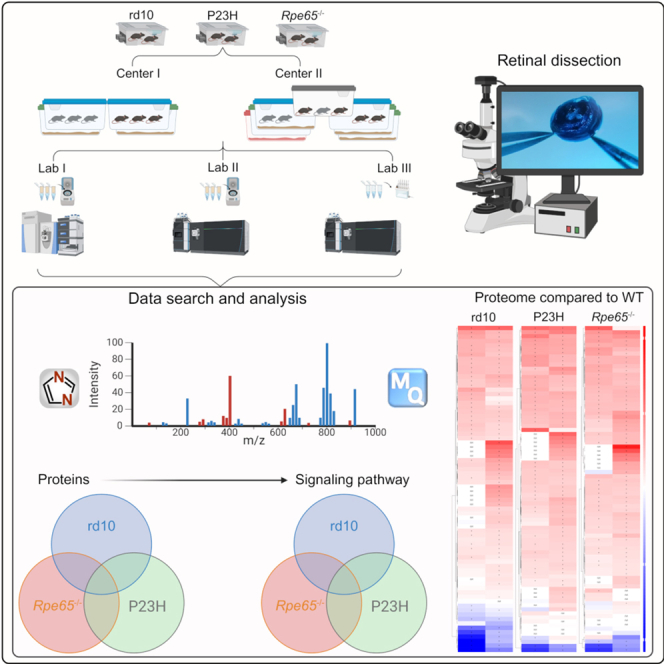

## In Brief

Inherited retinal degenerations (IRDs) lack effective treatment options and they are a major cause of blindness. In this study, we analyzed the retinal proteomes of three translationally relevant IRD mouse models (two for retinitis pigmentosa and one for congenital amaurosis type 2) to identify convergent signaling pathways using global proteomics. Despite different disease mechanisms between the models, we found shared pathways that could be targeted for broad therapeutic applications potentially by using already existing drugs.

Inherited retinal degenerative diseases (IRDs) are eye diseases that lead to significant disability and eventually to blindness, as they lack effective treatments. Collectively, IRDs are relatively common disorders with a global prevalence of 1/2000 (1), and they form a leading cause of blindness amongst the youth and working-age population in the developed world (2). IRDs are excellent targets for gene therapy as typically a single genetic mutation is the causative factor (3). Furthermore, local delivery of therapeutic agents in different compartments of the eye is common practice in eye clinics. These facts facilitated the clinical approval of the first gene therapy in 2017/2018 for the treatment of Leber's congenital amaurosis type 2 (LCA2), a severe type of IRD. This pioneering gene therapy, voretigene neparvovec (Luxturna), is a gene-augmentation therapy that supplies some normal RPE65 protein, which is crucial for the renewal of visual pigments in the retinal pigment epithelium (RPE) (4). Since mutations in *RPE65* account for only ∼1 to 2% of IRD cases (5, 6) and all affected patients are not eligible for Luxturna, a great majority of IRD patients are left without treatment options.

A major challenge with IRDs is that they are very heterogeneous genetically. To date, mutations in more than 280 genes are causative for IRDs, and the types of mutations in causative genes can be numerous (7). For instance, at least 150 different disease-causing mutations have been identified in the rhodopsin gene associated with retinitis pigmentosa (RP) (8). Although the genetic knowledge of IRDs is expanding constantly, 30% to 50% of IRDs remain idiopathic (9, 10). Because of these reasons, the applicability of targeted therapies to address all IRDs appears unlikely in the foreseeable future.

Even though the causative genetic mutations among IRDs are highly diverse, the pathophysiological signaling events occurring downstream of the primary insult (typically rod degeneration) could be shared. Such convergent pathological mechanisms, potentially driving collateral degeneration (11), are attractive targets for disease-modifying therapies (DMTs) that could benefit a broad and diverse patient population (12, 13, 14). However, a comprehensive picture of the pathological events across distinct IRD etiologies is still missing. Such data is crucial for enabling the rational design of DMTs. For instance, no cross-etiology analyses of the global proteome exist, although technically this would have been possible for at least a decade (15, 16).

In this study, we aimed to find convergent downstream retinal signaling events using global proteomics analyses from the retinas of three distinct IRD model mice. Specifically, we analyzed retinal samples from *Pde6β*^Rd10^ and *Rho*^P23H/WT^ (in short, rd10 and P23H) mouse models of autosomal recessive and autosomal dominant retinitis pigmentosa (RP), respectively; and samples from the *Rpe65*^−/−^ mouse model of LCA2. As controls, we analyzed retinal samples from age- and sex-matched wild-type (WT) mice corresponding to the respective IRD models. The mouse models in this work are highly utilized disease models in translational IRD research and they recapitulate the human diseases remarkably well (17). We found that the great majority of pathological proteomic changes were the same in rd10 and P23H RP-model mice, whereas in *Rpe65*^−/−^ LCA2-model mice numerous proteomic network changes were distinct. We focused our attention on the RP models and analyzed datasets not only by following the traditional LC-MS/MS Data-Dependent Acquisition (DDA) mode but also by using the Data-Independent Acquisition (DIA) mode for improved sensitivity. This study is the first one to analyze global proteomic data from several IRD models simultaneously. Our analysis identifies numerous convergent signaling pathways within the common RP models, facilitating the rational design of DMT strategies.

## Experimental Procedures

### Animal Models and Study Design

We used three different mouse models of inherited retinal degenerative diseases (IRD) to discover retinal proteomic changes that are common to retinal degeneration (RD). The models used in this study were B6.CXB1-*Pde6b*^rd10^/J (RRID: IMSR_JAX:004,297, referred to as rd10), B6.129S6(Cg)-*Rho*^tm1.1Kpal^/J (RRID: IMSR_JAX:017,628, referred to as P23H), mouse models of recessive and autosomal dominant retinitis pigmentosa (RP), respectively (18, 19), and B6.129-*Rpe65*^tm1Tmr^/J (RRID:IMSR_JAX:035,329, referred to as *Rpe65*^−/−^) model of Leber congenital amaurosis type 2 (LCA2) which was a kind gift from Dr Michael Redmond (National Institutes of Health) (20). The rd10 colony was kept as a homozygote. Age- and sex-matched C57BL/6J mice (RRID: IMSR_JAX:000,664) were used as controls. P23H heterozygote mice were bred with C57BL/6J mice yielding P23H heterozygote and wild-type (WT) littermates. To get *Rpe65*^−/−^ and their WT littermate mice, we bred heterozygote *Rpe65*^+/−^ mice together. Only WT and homozygote offsprings were used in this study. Several cohorts of mice were raised, and their retinal samples were collected, at two different institutions: the University of California Irvine (UCI) and the University of Eastern Finland (UEF) over the years 2019 to 2021 and 2023, respectively (Table 1). Mice were given water and standard feed *ad libitum* at both institutions. Retinal proteome analysis was conducted using liquid chromatography-tandem mass spectrometry (LC-MS/MS), employing data-dependent acquisition (DDA) or data-independent acquisition (DIA) modes.

**Table 1:** Age and housing conditions of mouse cohorts used in the study

Condition	DDA_rd10_DR	DDA_rd10_CLR	DDA_P23H	DDA_*Rpe65*^−/−^	DIA_rd10_DR	DIA_P23H
Disease model	RP	RP	RP	LCA	RP	RP
Age	P37	P38	P90	P45	P23–25	P60
Housing place	UCI	UCI	UCI	UCI	UEF	UEF
Rearing condition	DR	DR-to-CLR^a^	Normal	Normal	DR	Normal
LC-MS/MS	UCI	UO	UCI	UCI	UEF	UEF
MS instrument	Orbitrap Lumos	Orbitrap QExactive	Orbitrap Lumos	Orbitrap Lumos	Orbitrap QExactive	Orbitrap QExactive

DDA, Data-dependent acquisition; DIA, Data-independent acquisition; DR, Dark rearing; CLR, Cyclic light rearing; RP, Retinitis pigmentosa; LCA, Leber congenital amaurosis; UCI, University of California, Irvine; UEF, University of Eastern Finland; MS, Mass spectrometry; UO, University of Ottawa.

a Mice reared P0-P29 in DR and in CLR (vivarium) P29-P38.

The rd10 mice carry a naturally occurring point mutation in the phosphodiesterase 6b (*Pde6β*) gene. This mutation leads to instability and dysfunction of PDE6 protein and phototransduction, increased free cGMP, subsequent opening of cGMP-gated channels, and increased Ca^2+^ influx into rods, resulting in robust rod photoreceptor degeneration (21). The rd10 mice are highly susceptible to the damaging effects of light and most of their rods die by post-natal day 24 (P24) if mice are reared in cyclic light rearing (CLR), or vivarium conditions (18). The disease progression is substantially slower if rd10 mice are reared in a dim-light environment, or in a dark room. Several cohorts of rd10 mice and C57BL6/6J wild-type (WT) control mice were housed in different light environments, and used for this study:

UCI_DDA_rd10_CLR_cohort 1: rd10 (N = 6; n = 3 females, n = 3 males) and WT (N = 6; n = 3 females, n = 3 males) were housed in darkroom between P0 and P28. At P29, mice were transferred to standard vivarium housing conditions (CLR = lights on 6:30 AM, and off 6:30 PM) at the UCI laboratory animal center (LAC). Mice were euthanized by cervical dislocation at P38 (9 days in vivarium), their eyes were enucleated, and their retinas were harvested.

UCI_DDA_rd10_DR_cohort 2: rd10 (N = 6; n = 4 females, n = 2 males) and WT (N = 4; n = 2 females, n = 2 males) were housed in a darkroom at UCI-LAC throughout their lifespan. The only light exposure they experienced was the dim red light required for daily husbandry. Mice were euthanized by cervical dislocation at P37 in a dark room, their eyes were enucleated, and their retinas were harvested.

UEF_DIA_rd10_DR_cohort 3: rd10 (N = 4; n = 2 females, n = 2 males) and WT (N = 4; n = 2 females, n = 2 males) were housed in a dim light environment at UEF-LAC throughout their lifespan. The mice were kept in a Scantainer, in which glass doors were covered with a darkening film, limiting light exposure to <0.01 lux inside the mouse cages. The rd10 and WT mice were euthanized in the same session with a 3 min exposure to CO_2_ and subsequent cervical dislocation at P23 and P25, respectively.

P23H mice harbor a proline to histidine mutation in codon 23 in the rhodopsin gene, which leads to early rhodopsin misfolding, mislocalization, and subsequent ER stress in their retinas (22). The mutation leads to gain-of-function pathology, and *Rho*^P23H/WT^ mice undergo an intermediately progressing rod degeneration whereby roughly half of their rods die by 3 months of age (23). Unlike with the rd10 mice, the housing light conditions do not distinctly affect the disease progression in *Rho*^P23H/WT^ (P23H) mice, and all experiments in P23H mice were performed in standard vivarium conditions. Two cohorts of P23H mice and WT control mice were used:

UCI_DDA_P23H_cohort 1: P23H mice (N = 10; n = 5 females, n = 5 males) and WT littermates (N = 8; n = 4 females, n = 4 males) were housed at UCI-LAC. All mice were euthanized by cervical dislocation at ∼ P90, their eyes were enucleated, and their retinas were harvested.

UEF_DIA_P23H_cohort 2: P23H mice (N = 10; n = 5 females, n = 5 males) and C57BL/6J WT control mice (N = 5; n = 2 females, n = 3 males) were housed at UEF-LAC. The P23H and WT mice were euthanized by cervical dislocation at P60 and P57, respectively. Their eyes were enucleated, and their retinas were harvested.

The *Rpe65*^−/−^ mice are fully deficient of RPE65 which is an enzyme that is necessary for the functioning of the classical visual cycle in the retinal pigment epithelium (RPE) (24). In *Rpe65*^−/−^ mice, the cone photoreceptors do not respond to light at all (25). They also die quickly after the opening of the eyes due to cone opsin mislocalization in the absence of 11-*cis*-retinal production and supply by the RPE (26). In contrast, rods die slowly in *Rpe65*^−/−^ mice and they retain some residual light responsivity despite the severe chromophore insufficiency. In our experiment, the *Rpe65*^*−/−*^ mice were housed in standard vivarium conditions and one cohort was used:

UCI_DDA_*Rpe65*^−/−^ cohort 1: *Rpe65*^−/−^ mice (N = 8; n = 4 females, n = 4 males) and C57BL/6J WT control mice (N = 9; n = 3 females, n = 6 males) were housed at UCI-LAC. Both retinas from two *Rpe65*^−/−^ mice were pooled during harvesting leading to replicates: N = 4; n = 2 females, n = 2 males. Similarly, retinas from three WT mice were pooled during harvesting leading to replicates: N = 3; n = 1 female, n = 2 males. All mice were euthanized by cervical dislocation at P45, their eyes were enucleated, and their retinas were harvested.

At both institutions, mice were housed in a temperature-controlled animal facility with a 12-h light/dark cycle and fed a standard rodent diet *ad libitum*. All procedures were conducted in accordance with the ARVO Statement for the Use of Animals in Ophthalmic and Vision Research. Animal subjects at UCI were treated in accordance with the NIH guidelines for the care and use of laboratory animals, and all experimental procedures have been approved by the Institutional Animal Care and Use Committee (IACUC, protocol #AUP-21-096). Animal experiments at UEF were conducted in accordance with the Directive 86/609/EEC for animal experiments, and FELASA Guidelines and Recommendations, and were approved by the Finnish Project Authorization Board, with protocol number ESAVI/26320/2021.

### Retina Dissection

Retina dissection was performed as previously described (23). Briefly, following euthanasia, the eyes were quickly enucleated, and the retinas were excised by performing three incisions starting from the optic nerve head and cutting toward the *ora serrata*, which allowed easy and quick separation of the retina from the rest of the eye cup. Anterior parts of the eye were discarded, whereas the retinas were transferred to 1.5 ml Eppendorf tubes and snap-frozen using liquid nitrogen, and then stored for later processing.

### *In Vivo* Phenotyping by Electroretinography

We tested retinal function by electroretinogram (ERG) recordings under anesthesia with ketamine (100 mg/kg) and xylazine (10 mg/kg). ERG recordings were performed with a Diagnosys Celeris ERG device (Lowell), as described previously (27, 28).

### Label-Free Protein Quantifications Following Data-dependent Acquisition Mode

UCI_DDA_rd10_DR_cohort 2, UCI_DDA_P23H_cohort 1, and UCI_DDA_Rpe65^−/−^ cohort one were analyzed following the method A steps, while UCI_DDA_rd10_CLR_cohort one was analyzed following method B steps (see below).

### Sample Preparation and Protein Digestion

#### Method A

The retinas were suspended in Urea buffer containing 8 M Urea, 0.1 M Tris-HCl (pH 8.5), 1% protease inhibitor cocktail, and ultrasonicated on ice for 4 min, followed by centrifugation at 12,000*g* at 4 °C for 10 min. The resulting supernatant (*i.e.*, extracted retinal proteins) was collected for digestion using the filter-aided sample preparation (FASP) method (29). In brief, the extracted retinal proteins were loaded into an Amicon centrifugal filter (Millipore) with a 30-kDa cutoff. The proteins were then reduced with 10 mM dithiothreitol (DTT) at 56 °C for 1 h and alkylated with 20 mM iodoacetamide (IAA) at room temperature (RT) in the dark for another 1 h. Subsequently, the buffers were replaced with 50 mM ammonium bicarbonate (AmBi) through three washes of the filter membrane. Modified trypsin (Promega) was added to the protein solution in a 1:50 (w/w) ratio and incubated at 37 °C overnight. The digested peptides were collected via centrifugation, along with an additional water rinse. This solution was then vacuum-dried and reconstituted in 200 μl of 0.5% acetic acid. The peptide mixture was desalted by C18 solid-phase extraction (provided by The Nest Group, Inc), and vacuum-dried. For each analysis, 0.5 μg of digested proteins were loaded for LC-MS/MS analysis in randomized order.

#### Method B

Frozen specimens were ground to a powdered state over liquid nitrogen. Fresh chilled sodium deoxycholate (SDC) lysis buffer (4 % wt/vol SDC and 100 mM Tris-HCl, pH 8.5) was then added, and the samples were incubated at 95 °C for 5 min with shaking at 1500 rpm. This was followed by sonication (Q125 Sonicator, Qsonica, LLC) at 4 °C at maximum output power for two 10-min cycles. The samples were reduced and alkylated using 10 mM TCEP and 40 mM 2-chloroacetamide (pH 7) (Sigma) while incubating for 5 min with shaking at 1500 rpm at 45 °C, and then cooled. Lys-C (Thermo Scientific, 90,051) and TPCK-treated trypsin (Worthington-biochem, LS02124) were added at an enzyme-to-substrate ratio of 1:100 (w/w) and digested for 16 h at 37˚C with shaking at 1500 rpm. The SDC was removed from the samples by acidification with formic acid (FA) to ∼ pH 2, followed by centrifugation at 16,000 g for 5 min. Next, 5 mg of a slurry of 10-μm C18 column beads (Dr Maisch GmbH) in acetonitrile (Sigma-Aldrich, 1000294000) was loaded into a 200 μl filter tip (Vertex, 4237NSF) and the tryptic peptides were washed three times with 0.1% FA (v/v). The peptides were then eluted with 80% acetonitrile (v/v) (Sigma-Aldrich, 1000294000) and 0.1% FA (v/v), freeze-dried, and reconstituted to 1 μg/μl with 0.5% formic acid (v/v) (Sigma-Aldrich, 5330020050). An aliquot (1 μl) of each sample was loaded for LC-MS/MS analysis in randomized order.

### Peptide Separation and Data-dependent Acquisition (DDA)

#### Method A

Proteomics analysis was performed using an UltiMate 3000 UHPLC system (Thermo Fisher Scientific) connected directly to an Orbitrap Fusion Lumos mass spectrometer (Thermo Fisher Scientific) equipped with an ESI nanospray source. The mobile phase A consisted of 0.1% FA in water, and mobile phase B consisted of 0.1% FA in ACN. The peptides were eluted at a constant flow rate of 300 nl/min and separated over an active 57-min gradient from 4% to 25% buffer B, with a total sample runtime of 90 min on an Acclaim PepMap RSLC column (50 cm × 75 μm). Survey MS scans were conducted in the Orbitrap (FT) with an automated gain control (AGC) target of 8E5, a maximum injection time of 50 ms, and a dynamic exclusion period of 30 s covering a scan range of 375 to 1800 m/z. MS/MS spectra were gathered in data-dependent acquisition (DDA) mode at the highest speed setting for 3-s cycles; the AGC target was set at 1E4 with a maximum injection time of 35 ms. The ions underwent stepped-energy, higher-energy collisional dissociation (seHCD) with a normalized collision energy (NCE) of 20 ± 5%.

#### Method B

Proteomics analysis was conducted using an EASY-nLC 1200 System (Thermo Fisher Scientific) linked to a Q-Exactive mass spectrometer (Thermo Electron) via a nano-electrospray interface operating in positive ion mode. Mobile phase A consisted of 0.1% FA in water, while mobile phase B was comprised of 0.1% FA in 80% acetonitrile. Peptides were loaded into a 75 μm I.D. × 150 mm fused-silica analytical column packed in-house with 3 μm ReproSil-Pur C18 resin (Dr Maisch GmbH). The flow rate was set at 250 nl/min, and peptides were separated over a 105-min active gradient from 5% to 35% buffer B (total method duration 120 min). Survey MS scans were captured in the Orbitrap with a resolution of 70k at m/z 400, and the spray voltage was held at 2.0 kV. The capillary temperature was maintained at 300 °C. Data-dependent MS/MS scans were performed targeting the 12 most intense precursor ions with a dynamic exclusion of 30 s. MS/MS resolution was set at 17.5k, and real-time internal calibration was used to enhance mass accuracy with a lock mass of background ion at 445.120025. Charge states that were unknown or singly charged were excluded from MS/MS analysis. All data were collected using Xcalibur software (ThermoFisher Scientific).

### DDA Spectrum Match and Identification of Proteins

The raw LC-MS/MS data files were processed using MaxQuant (version 2.1.0.0) (15), with the spectra matched against the Uniprot mouse database (UP000000589, updated in April 2022, which contains 21,957 protein entries plus additional Uniprot mouse database which contains 41,543 protein isoform entries) and cRAP contaminant database (updated in March 2019, which contains 100 protein entries). For peptide identification, the mass tolerances applied were 20 ppm for initial precursor ions and 0.5 Da for fragment ions. Specific Trypsin/P digestion and up to two missed cleavages were permitted in the tryptic digests and spectral search. Cysteine residues were treated as static modifications, and oxidation of methionine was considered a variable modification. Peptide identification filtering was conducted at a 1% false discovery rate.

### Label-Free Quantification of Proteins Following Data-Independent Acquisition Mode

UEF_DIA_rd10_DR_cohort 3, and UEF_DIA_P23H_cohort two were analyzed following method C steps.

### Sample Preparation and Protein Digestion

#### Method C

The mouse retinas were homogenized in 100 μl of protein extraction buffer (ab193970) (Abcam) using a handheld homogenizer (Pellet Pestle Cordless Motor, FisherScientific) for 30 s on ice. The retinal homogenates were then solubilized by sonication in two 30-s bursts, interspersed with 30-s intervals on ice. The lysates were then centrifuged at 18,000*g* for 20 min at 4 °C. The supernatants (extracted solubilized proteins) were collected in separate Eppendorf low-protein binding tubes (Thermo Fisher Scientific). Total protein content was measured from retinal lysates using the BCA protein assay (Thermo Fisher Scientific), and the total protein concentration was adjusted to 1 mg/ml for all samples using the same lysis buffer. The extracted and solubilized retinal proteins were processed using FASP, as previously described (29, 30). Briefly, a total of 50 μg of protein fraction was loaded on a centrifugal filter 30-kDa cutoff (Merk Millipore), and the buffer was exchanged with 0.1 M DTT (Merk) in UA buffer (8 M Urea in 0.1 M Tris/HCl, pH 8.5). The mixture was incubated at RT on a thermomixer at 500 rpm for 60 min and then washed twice with UA buffer. Alkylation of the protein samples was performed using 0.05 M iodoacetamide (Merck) in UA buffer on the thermomixer at 300 rpm for 20 min in the dark. The reduced and alkylated proteins on the filter were washed twice with UA buffer, then incubated with 49 μl of 50 mM AmBi digestion buffer, 1:100 (w/w) endoproteinase LysC, and 0.05% ProteaseMax (Promega) on the thermomixer at 600 rpm and 30 °C for 3 h. Subsequently, 1 μl of TPCK-treated trypsin (Promega) was added to the filter at a 1:50 (w/w) ratio and incubated for 16 h at 37 °C. The digested peptides were recovered by centrifugation followed by two elution steps using 50 μl of 50% acetonitrile in AmBi buffer. The solvent was evaporated via a SpeedVac vacuum concentrator (Thermo Fisher Scientific) at RT. The dried samples were reconstituted in a 2% acetonitrile/5% FA solution, and mixed on a thermomixer at 300 rpm and RT for 20 min. Finally, 10 μl of each sample, containing 10 μg of protein, was loaded for LC-MS/MS analysis in randomized order.

### Peptide Separation and Data-independent Acquisition (DIA)

Proteomics analysis was conducted using a UPLC (Vanquish Flex, Thermo Scientific) coupled to a high-resolution Orbitrap Q Exactive Classic mass spectrometer (Thermo Scientific) operating in positive ion mode. Mobile phase A consisted of 0.1% FA in water, while mobile phase B comprised 0.1% FA in ACN. Peptides were loaded into an Agilent AdvanceBio Peptide Map column (2.1 mm × 250 mm, 2.7 μm, Agilent Technologies). The flow rate was set at 0.3 ml/min, and peptides were separated over an 80-min active gradient from 2% to 45% buffer B (total method duration 90 min). Mass spectrometric detection encompassed Full MS–SIM (Resolution: 35k; AGC target: 3e6; max injection time: 60 ms; scan range: 385–1015 m/z) and DIA (Resolution: 17.5k; AGC target: 2e6; max injection time: 60 ms; loop count: 25; isolation window: 24 m/z).

### DIA Spectrum Match and Identification of Proteins

The raw data were processed by DIA-NN software (version 1.8) using the library-free DIA analysis mode (31) with default settings. The MS/MS spectra library and retention times of peptides were predicted using the UniProt reference proteome database for mouse (UP000000589, updated in April 2022, which contains 21,957 protein entries plus additional Uniprot mouse database which contains 41,543 protein isoform entries). Cysteine residues were set as static modifications. Oxidation of methionine and N-terminal acetylation were set as variable modifications while a maximum number of variable modifications per peptide was set to 2. The predicted MS library was used to search the raw data, applying 1% thresholds for both precursor and protein group false detection rates, and requiring the presence of at least one proteotypic peptide ranging from 7 to 30 amino acids in length. For data evaluation, the resulting MaxLFQ normalized intensities (15) were used.

### Experimental Design and Statistical Rationale

The output protein groups and their normalized intensities, calculated by the MaxLFQ algorithm (15) integrated into Maxquant and DIA-NN software, were used for further downstream analysis. The number of replicates used in each mouse cohort is described in Table 2, while detailed sample characteristics are described in the supplementary file (Supp4-Sample_characteristics).

**Table 2:** Number of replicates used for retinal proteome analysis

Condition	DDA_rd10_DR	DDA_rd10_CLR	DDA_P23H	DDA_*Rpe65*^−/−^	DIA_rd10_DR	DIA_P23H
RD model	6 (4f + 2m)	6 (3f + 3m)	7 (2f + 5m)	4 (2f + 2m)^a^	4 (2f + 2m)	10 (5f + 5m)
WT	4 (2f + 2m)	6 (3f + 3m)	8 (4f + 4m)	3 (1f + 2m)^b^	4 (2f + 2m)	4 (1f + 3m)

RD: retinal degeneration, WT: wildtype, f: female, m: male.

a both retinas of two mice were pooled per replicate.

b single retina of three mice were pooled per replicate.

**Fig. 1:**
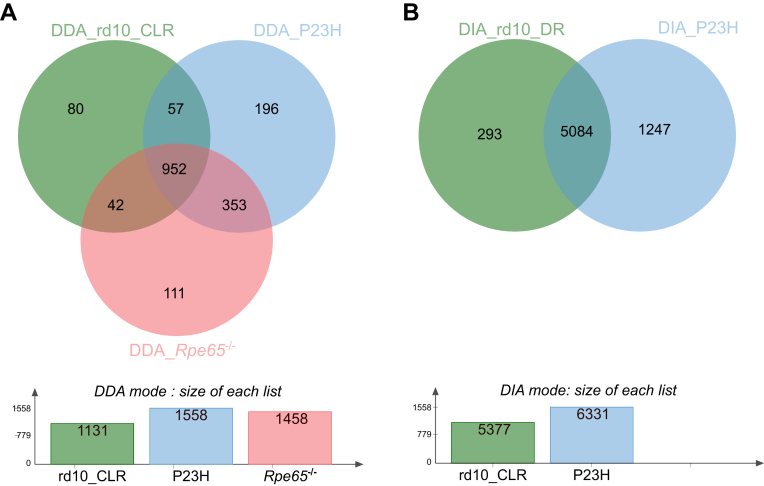
**Comparison of the total number of reliably identified and quantified proteins between the DDA and DIA analysis modes**. The criterion for reliable identification: at least three valid values per group were required. *A*, DDA analysis mode; DDA_rd10_CLR cohort (rd10, n = 6; WT, n = 6), DDA_P23H (P23H, n = 10; WT, n = 8), DDA_*Rpe65*^−/−^ cohort (*Rpe65*^−/−^, n = 4; WT, n = 3). *B*, DIA analysis mode; DIA_rd10_CLR cohort (rd10, n = 4; WT, n = 4) and DIA_P23H cohort (P23H, n = 10; WT, n = 5). The DDA spectral data were deconvoluted with the Andromeda search engine integrated into MaxQuant software, while the DIA spectral data were deconvoluted using DIANN software. Both datasets were filtered at a 1% FDR identification rate. CLR, Cyclic light rearing; DR, Dark rearing; WT, wildtype.

The MaxLFQ intensities were filtered and processed further using Perseus software (32). First, potential contaminants, only identified by site, or reversed hits were filtered out. The intensities of the protein groups were log-transformed, and the study groups were defined using the category-annotation tab. The groups were then divided based on data completion and missing values into two groups; complete dataset (100% valid values in total) and missing values dataset (at least three valid values in at least one group). The categorical groups in the complete datasets were compared and analyzed using a linear model for microarray data test (LIMMA) (33) followed by multiple testing false discovery rate (FDR) corrections; and the statistical significance was set at *q-value* < 0.05. The categorical groups in the missing values datasets were analyzed using the Student’s *t* test and the statistical significance was considered if there were at least three valid values in each of the compared groups and a *p-value* < 0.01. The KEGG (Kyoto Encyclopedia of Genes and Genomes) pathway analysis from statistically significant differentially expressed proteins (DEPs) was performed using SRplot (34). Data visualization was performed using several R packages such as dplyr (35), ggplot2 (36), KEGGREST (37), LIMMA (33) pheatmap (38), and VennDiagram (39). Figure 1 and Supplemental Figs. S1 and S2 were created using Jvenn (40), while violin plots were prepared by SRplot (34). Inkspace 1.2 software was used to combine the figures.

## Results

### Profiling of the Mouse Retinal Proteome

In the present study, we identified and quantified over 7000 mouse retinal proteins from 6 mouse cohorts raised at two different institutions, UCI and UEF. Frozen retinal samples were processed in three different LC-MS laboratories (at UCI, UO, and UEF) using two different label-free protein quantification methods, the DDA and DIA modes. Consistent and reliable identifications are presented in (Fig. 1).

The standard data-dependent acquisition (DDA) LC-MS mode was employed to analyze the retinal proteome of three different IRD mouse models (rd10, P23H, and *Rpe65*^−/−^). Roughly 53% of identified retinal proteins were quantified in all three models (Fig. 1*A*). To investigate the RP-associated retinal proteome more comprehensively, we employed the DIA analysis mode through the spectral library prediction tool, as previously described (31). Importantly, by using the DIA mode the overall proteome coverage was improved more than four-fold (Fig. 1*B* and Supplemental Figs. S1 and S2). In the DIA analyses, 77% of identified proteins were quantified in both RP models.

Nevertheless, the consistent identification of proteins across various research sites and analytical methods reinforces the reliability and potential for comparative (semi-quantitative) analysis between the datasets (Supplemental Fig. S4). Indeed, selected marker proteins showed a similar pattern of expression change regardless of mouse housing institution (UCI & UEF), mass spectrometer used, or analysis mode (DDA & DIA) used in dark-reared rd10 mice (Supplemental Fig. S4*A*) or vivarium-housed P23H mice (Supplemental Fig. S4*B*). Instead, laboratory light conditions affected the retinal phenotype of the rd10 mouse significantly (Supplemental Fig. S5), which has also been described before (41).

### Phenotypes of the Three IRD Models Based on Electroretinography Recording and Marker Protein Expression

**Fig. 2:**
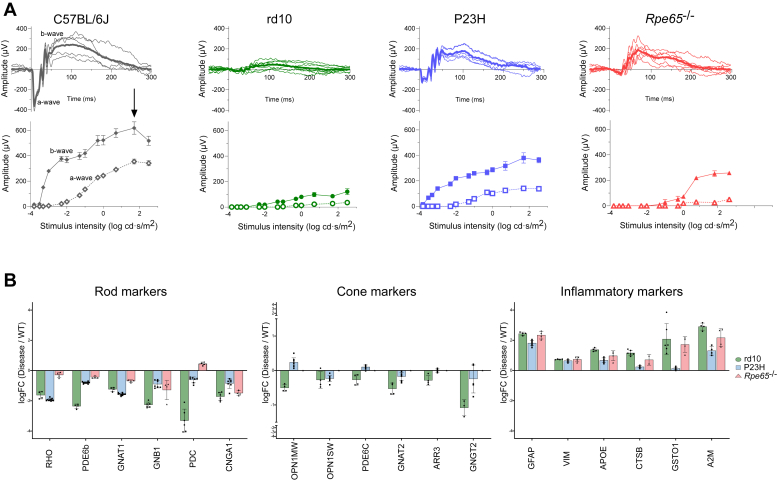
**Phenotypic characterization of the three IRD models used in DDA-mode proteomics.** Phenotyping is based on scotopic ERG recordings and the expression of cell-enriched marker proteins. *A*, *upper panels* show scotopic ERG waveforms (*thick line* represents group-mean responses, while thin lines present individual mice) in response to an amplitude-saturating stimulus (log 1.7 cd s/m^2^). *Lower panels* present ERG a- and b-wave amplitudes across a stimulus-intensity series. *Gray*, WT, n = 5; *green*, rd10, n = 7; *blue*, P23H, n = 5; *red*, *Rpe65*^−/−^, n = 7. *B*, protein expression changes expressed as log2FC values on the y-axis across three IRD mouse models compared to their respective WT littermates, showing selected retinal rod, cone, and inflammatory markers. The data are presented as mean ± SEM.

**Fig. 3:**
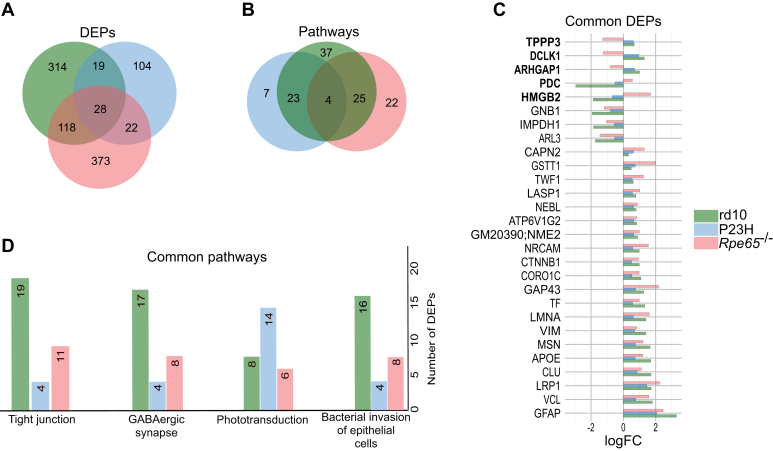
**Retinas from rd10, P23H and *Rpe65***^***−/−***^**mice display only a handful of common DEPs.***Green*, Rd10; blue, P23H; red, *Rpe65*^*−/−*^. *A*, The Venn diagram shows the number of common and specific DEPs. *B*, the Venn diagram shows the number of common and specific enriched KEGG pathways across the three IRD models. *C*, the bar graph shows the common DEPs across the three models and their expression, as depicted by their log2FC compared to their respective WT mice. *D*, the bar graph shows all the common enriched KEGG pathways across the three models.

**Fig. 4:**
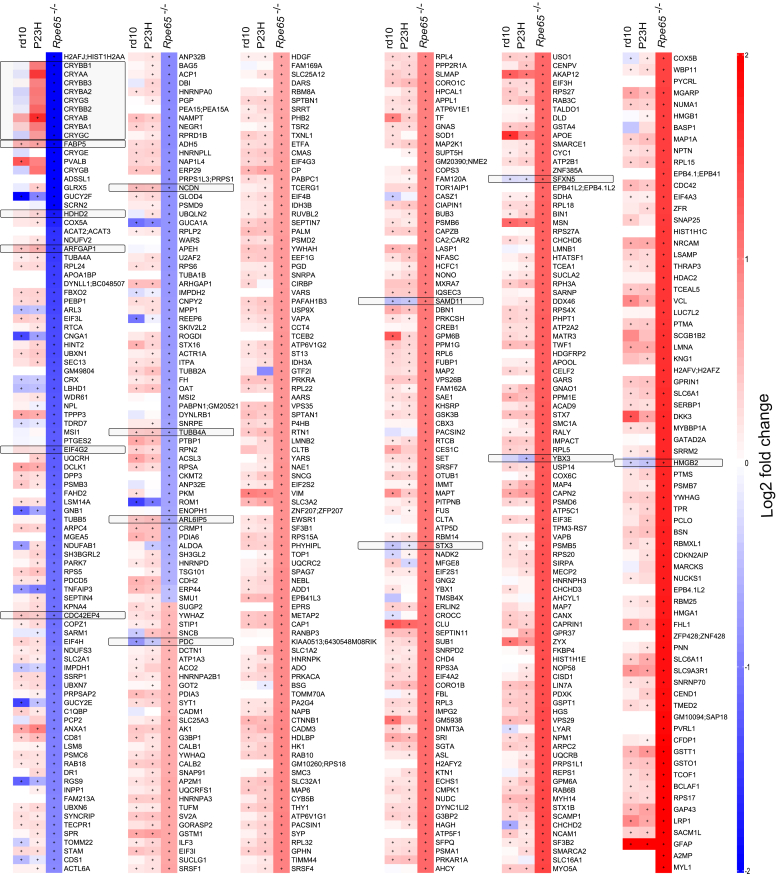
**The altered retinal proteome of LCA2-*Rpe65***^***−/−***^**mice is largely different from those of RP mice.** Heatmap focusing on the log2FC of the DEPs in retinas of *Rpe65*^−/−^ mice (*Rpe65*^−/−^, n = 4; WT, n = 3). Expression of the same proteins is shown for the rd10 (rd10, n = 4; WT, n = 4) and P23H models (P23H, n = 10; WT, n = 5). Statistical analysis was performed by the LIMMA test followed by FDR correction. The + signs indicate statistically significant changes at *q-value* < 0.05. DEPs that show opposing directions of regulation between the *Rpe65*^*−/−*^ and RP models are highlighted with boxes.

**Fig. 5:**
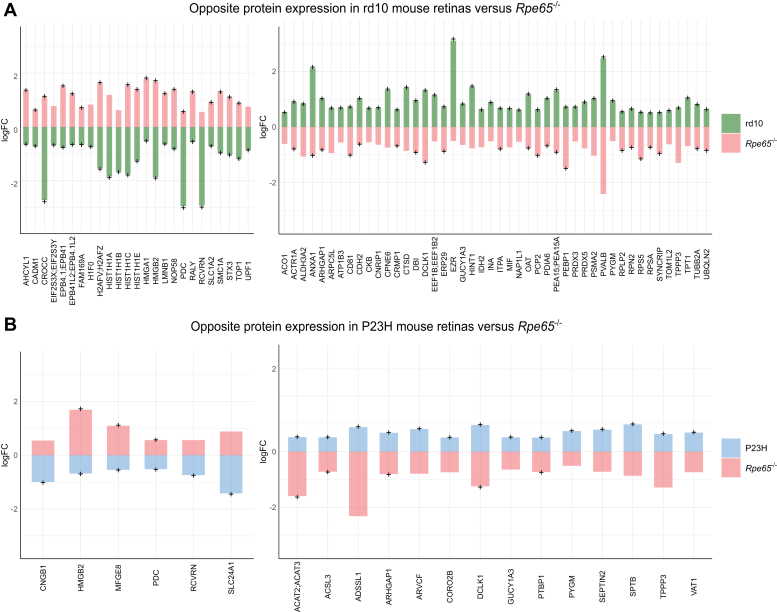
**Many proteins show differential direction of expression change in *Rpe65***^***−/−***^***versus* rd10, or *Rpe65***^***−/−***^***versus* P23H mouse retinas.** DEPs (based on LIMMA test followed by FDR correction with parameters: *q-value* < 0.05 and log2FC > 0.5) in the retinas of rd10 mice (graph *A*; rd10, n = 6; WT, n = 6), or P23H mice (graph *B*; P23H, n = 10; WT, n = 8) as compared to those of *Rpe65*^−/−^ mice (*Rpe65*^−/−^, n = 4; WT, n = 3) are plotted on the x-axis, and the amplitude of log2FC (disease model *versus* WT) is plotted on the y-axis. Proteins selected for this comparison needed to be statistically significant DEPs in the retinas of at least the rd10 or P23H mice; but statistical significance (*q-value* < 0.05) is also marked with + signs for the retinas of *Rpe65*^*−/−*^ mice.

**Fig. 6:**
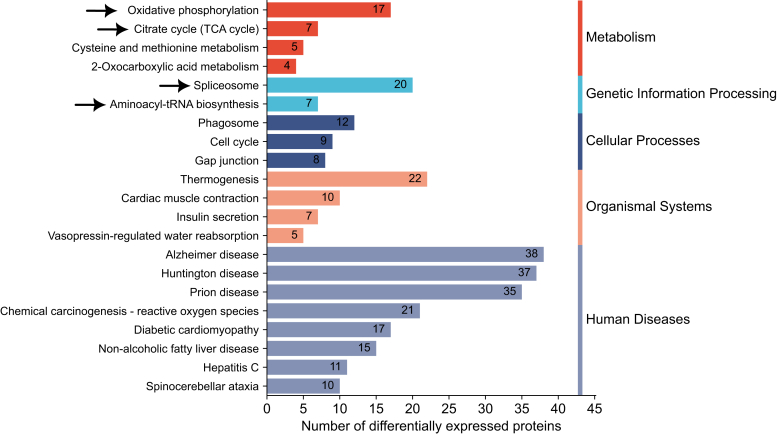
**Several KEGG pathways are uniquely altered in Rpe65^−/−^ mouse retinas.** KEGG pathways enriched exclusively in the retinas of Rpe65^−/−^ showing the number of DEPs involved in each pathway. Statistical analysis was performed by LIMMA test followed by FDR corrections. DEPs here were identified with parameters: q-value < 0.05 and log2FC > 0.5, compared to protein expression of their respective WT retinas. The arrows point to pathways of specific interest.

**Fig. 7:**
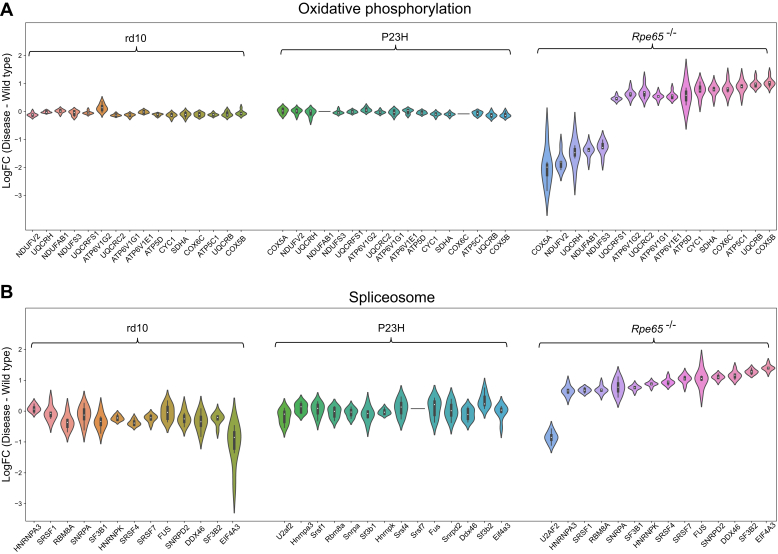
**Many key components of the oxidative phosphorylation and spliceosome system are uniquely altered in Rpe65**^**−/−**^**mouse retinas.** Many key components of the oxidative phosphorylation (*A*) or spliceosome (*B*) system are uniquely altered in *Rpe65*^*−/−*^ mouse retinas. The proteins highlighted in this figure are DEPs in the *Rpe65*^*−/−*^ mouse retinas (*Rpe65*^−/−^, n = 4; WT, n = 3), and their expression change is contrasted to the expression change of rd10 (rd10, n = 6; WT, n = 6) and P23H (P23H, n = 10; WT, n = 8) retinas. Statistical analysis was performed by LIMMA test followed by FDR multiple testing corrections. DEPs were identified as differentially expressed proteins with the following parameters *versus* WT: *q-value* < 0.05 and log2FC > 0.5.

Although the phenotypes of rd10, P23H, and *Rpe65*^−/−^ mice are well-characterized in the literature (see *e.g.*, (18, 19, 20), we recorded scotopic electroretinograms (ERG) to highlight some major differences between the models (Fig. 2*A*). At the time of ERG recording, the mice were housed in similar conditions and were at similar ages as those used for the DDA mode-based proteomics (DDA proteomics data presented in Fig. 3, Fig. 4, Fig. 5, Fig. 6, Fig. 7). The ERG responses in rd10 mice at this stage (P39) were very small (Fig. 2*A*), and likely dominated by the activity of surviving cone photoreceptors (42). In contrast, the P23H mice (P90) displayed intermediately well-preserved and sensitive ERGs, particularly with respect to the b-wave (Fig. 2*A*). These responses are driven by the remaining rods together with the well-preserved cone population (23). The *Rpe65*^−/−^ mice (P42) displayed a minuscule a-wave but surprisingly strong b-wave amplitudes (Fig. 2*A*). It is notable, however, that the *Rpe65*^−/−^ mice only started to respond at the highest stimulus intensities, which indicates low sensitivity. These unusual light responses of the *Rpe65*^−/−^ mice are known to arise from residual rod activity (43). In fact, retinal light responses are severely attenuated from birth in *Rpe65*^*−/−*^ mice (25), and they may be practically blind at low light levels such as in standard vivarium conditions.

For proteomic data processing, before moving into analysis of DEPs and KEGG pathways, we selected several rod- and cone-enriched markers (44) (Supplemental Fig. S3), as well as common inflammation markers, to provide an estimate of how the different models compare with respect to photoreceptor degeneration and inflammatory status at the time of sample collection. Analysis of rod-enriched markers indicated a significant loss of rod photoreceptors, with rd10 being the most affected model, as evidenced by the decreased expression of rod-enriched proteins (Fig. 2*B*). The pronounced downregulation of RHO and GNAT1 was comparable in rd10 and P23H models. Rod degeneration is relatively slow in *Rpe65*^*−/−*^ mice (26), which is evident in our data (Fig. 2*A*).

Analysis of cone-enriched markers OPN1MW, OPN1SW, PDE6C, GNAT2, and GNGT2 showed downregulation collectively only in the rd10 mice, but not in P23H mice (Fig. 2*B*). None of the cone-enriched markers were detected in *Rpe65*^*−/−*^ mice (Fig. 2*B*), highlighting the severe anatomic cone-degeneration in these mice (45). Interestingly, while the short-wavelength sensitive S-opsin (OPN1SW) was downregulated in P23H mice also, the middle-wavelength sensitive M-opsin (OPN1MW) showed upregulation. This finding suggests that the S-cones may be more susceptible to cell death in the *P23H* mice, or that retinal degeneration overall is more pronounced in the inferior retina where these cells are predominantly expressed (46). The trend towards increased OPN1MW expression in P23H retinas could be due to homeostatic regulation to counterbalance rod-pathway dysfunction. Indeed, we previously observed oversensitive cone-mediated ERG b-wave responses in P23H mice up to 3 months of age (28). In the same study, S-cone responses appeared to start declining earlier than M-cone responses.

Inflammation markers GFAP, VIM, APOE, CTSB, GSTO1, and A2M indicated significant inflammatory response in the retinas of all three IRD models (Fig. 2*C*). Overall, the order of inflammatory status in the models at this disease stage appeared to be from highest to lowest: rd10, *Rpe65*^−/−^, and P23H. Table 3 provides a qualitative summary of retinal degeneration severity in the three models.

**Table 3:** Summary of disease model phenotype

Model	rd10	P23H	*Rpe65*^−/−^
Primary associated disease	RP	RP	LCA
Rod degeneration progression	+++	++	+
Cone degeneration progression	++	+	+++
Combined rod-cone dysfunction (ERG)	+++	++	+++
Inflammation markers	+++	++	+++

Analysis derived from literature data, as well as ERG and marker protein expression data from this work.

### Commonly Regulated Retinal Proteome in the RP and LCA Mouse Models

The retinas from the three IRD models shared only a few tens of commonly regulated DEPs (Fig. 3*A*), leading to a handful of KEGG pathways that were commonly enriched among the models (Fig. 3*B*). The common DEPs largely consisted of cell stress-related proteins such as VIM and GFAP (Fig. 3*C*). The four commonly enriched KEGG pathways were: “Tight junction”, “GABAergic synapse”, “Phototransduction”, and “Bacterial invasion of epithelial cells” (Fig. 3*B*). Downwards regulation in the “Phototransduction” pathway (Supplemental Fig. S6) is clearly evident, as a majority of its components are expressed in the photoreceptor cilia that stereotypically degenerate in IRDs, including in the disease models used here. The “Tight junction” pathway tends to be upregulated in all three models, and commonly regulated proteins include several actin- and myosin-related proteins such as EZR, MYH9, MSN, ACTN1, and SLC9A3R1. These changes may act to improve cell mobility, contractility, polarity, and survival. The regulation mediated by the KEGG pathway “Bacterial invasion of epithelial cells” is also largely linked to changes in cytoskeleton-related components such as actin, catenin, and filament-forming proteins. The overall upregulation of “GABAergic pathway” proteins (Supplemental Fig. S7) indicates modified GABA activity.

### Selectively Regulated Retinal Proteome in the LCA Model Versus the RP Models

A total of 373 DEPs were dysregulated in *Rpe65*^*−/−*^ mice compared to WT (Fig. 4 and Supp2-proteins list). Among these proteins, 83 showed expression changes in the opposite direction (either upregulation or downregulation) compared to those seen in rd10 or P23H mice, as illustrated in (Fig. 5). Many crystallin proteins were highly downregulated in *Rpe65*^*−/−*^ samples while being stable or slightly upregulated in the RP mouse retinas (Fig. 4). Similarly, some proteins related to nucleotide metabolism (ARFGAP1, ARL6IP5, & CDC42EP4), or cytoskeleton (EIF4G2, HDHD2, & TUBB4A), were downregulated in *Rpe65*^−/−^, but trending towards upregulation in RP mice. Fatty acid-binding protein 5 (FABP5) was distinctly downregulated in *Rpe65*^−/−^ mouse retinas (>−2 log2FC), while being upregulated in rd10 and P23H mice (>0.5 log2FC). Neurochondrin (NCDN) also followed the same pattern. In contrast, some proteins such as PDC, SAMD11, STX3, SFXN5, YBX3, AND HMGB2 were upregulated in *Rpe65*^*−/−*^ mice, but downregulated in both rd10 and P23H mice.

There was more discrepancy in *Rpe65*^*−/−*^ *versus* rd10 mouse proteomic regulation, as compared to *Rpe65*^*−/−*^ versus P23H (Fig. 5). For instance, proteins related to nucleosome and chromosome phasing and histone binding to DNA (*e.g.*, H1F0, H2AFV; H2AFZ, HIST1H1A, HIST1H1B, HIST1H1C, HIST1H1E, HMGA1, LMNB1, NOP58, RALY, SMC1A, TOP1, and UPF1) were upregulated in *Rpe65*^*−/−*^ but downregulated in rd10 mouse retinas, while this pattern was absent in P23H retinas.

The proteomic phenotype of *Rpe65*^*−/−*^ mouse retina had several distinct features (Fig. 6). These included dysregulation in oxidative phosphorylation, the citrate cycle (TCA), and the spliceosome pathway. Several components of the mitochondrial respiratory chain complex I (NDUFV2, NDUFAB1, NDUFS3), also known as the NADH: ubiquinone oxidoreductase, were significantly downregulated in *Rpe65*^*−/−*^ mouse retinas (Fig. 7*A*). Instead, a major component of respiratory chain complex II, SDHA, was significantly upregulated. Several components of the downstream complexes III and IV showed bidirectional regulations (*e.g.*, opposite regulation of UQCRH and UQCRB, or COX5A and COX5B); whereas the subunits of complex V, or ATP synthase (such as ATP5D and ATP5C1), collectively were upregulated in the *Rpe65*^*−/−*^ samples. The protein expression in the components mentioned above remained stable in both RP models. Tens of proteins included in the TCA cycle pathway also showed bidirectional regulation in *Rpe65*^*−/−*^ retinas, but their regulation remained collectively more stable in rd10 and P23H retinas (Supplemental Fig. S8). Interestingly, while aconitase 1 (ACO1) and mitochondrial isocitrate dehydrogenase (IDH2) were clearly downregulated in *Rpe65*^*−/−*^ retinas, the same proteins were distinctly upregulated in rd10 retinas.

Changes in the proteome of the *Rpe65*^*−/−*^ retina also suggested an altered spliceosome pathway through overall upregulation of spliceosome components, such as ribonucleoproteins (HNRNPK, HNRNPA3, SF3B2, & SNRPD2), RNA helicases (DDX46 & EIF4A3), and other regulatory or RNA-binding proteins (FUS, SF3B1, SNRPA, RBM8A) (Fig. 7*B*). This phenotype was not evident in the RP mouse retinas.

### Analysis of the Retinal Proteome of the RP Mouse Models with Increased Coverage

**Fig. 8:**
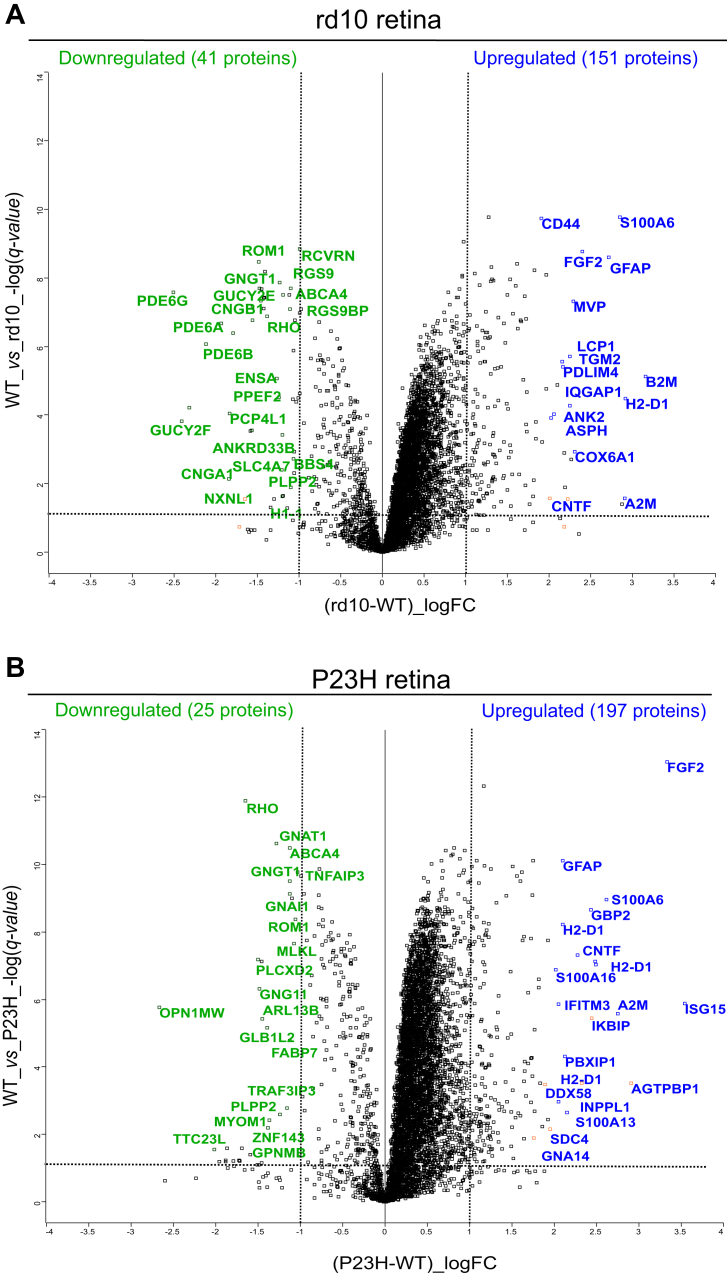
**Pathologic upregulation of proteins is more prevalent than downregulation in RP retinas.** The names of DEPs with largest expression change (−1 < log2FC > 1) in rd10 (rd10, n = 4; WT, n = 4) (*A*) or P23H (P23H, n = 10; WT, n = 5) (*B*) mouse retinas are marked in the volcano plots. Log2FC is plotted on the *x*-axis with cut-off values at ±1 (*vertical dashed lines*), while −log *q-value* is plotted on the *y*-axis with the cut-off value at 0.05 (*horizontal dashed line*). The statistical analysis was performed by the LIMMA test followed by FDR multiple testing corrections.

The DIA-MS-based analysis of the retinal proteome for rd10 and P23H mice showed higher proteome coverage compared to the earlier DDA-MS-based method (∼four-fold increase) (Fig. 3 and Supplemental Fig. S2). This increased coverage enabled a more detailed analysis of the proteome of the two RP models. As a distinct general feature, significantly upregulated DEPs (*q-value* < 0.05 and log2FC > 1) greatly outnumbered downregulated DEPs (Fig. 8). A total of 151 and 197 proteins were upregulated compared to only 41 and 25 downregulated proteins in the rd10 and P23H retinas, respectively (Fig. 8, *A* and *B*). As expected, due to photoreceptor degeneration, most distinctly downregulated proteins included photoreceptor cilia-specific components such as RHO, ROM1 and subunits of PDE6. The most strongly upregulated proteins in both RP models included immune system/inflammation/stress response-associated proteins such as GFAP, FGF2, H2-D1, S100 A.

**Fig. 9:**
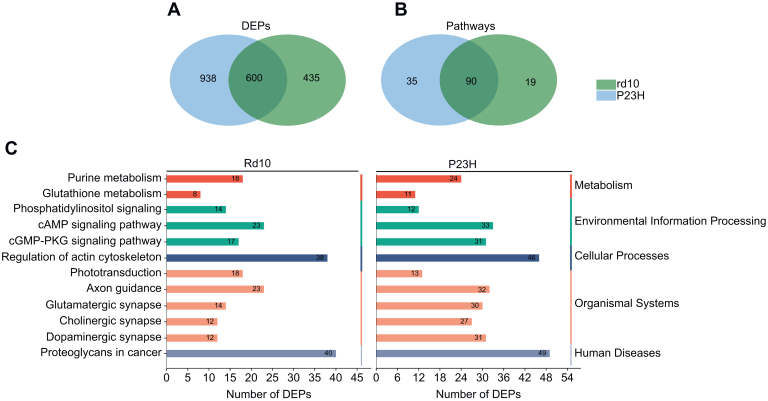
**High-coverage retinal proteomics of rd10 and P23H mouse models of RP reveal several hundreds of commonly regulated DEPs.** The Venn diagrams show the number of DEPs (*A*) and enriched KEGG pathways (*B*) that are common in the rd10 and P23H retinal proteomes. The bar graphs (*C*) show selected KEGG pathways with the number of involved proteins in each enrichment. Statistical analysis was performed by the LIMMA test followed by FDR multiple testing corrections. DEPs were identified according to the following parameters: *q-value* < 0.05 and log2FC > 0.5.

Both rd10 and P23H retinas share 600 DEPs (Fig. 9*A*) which form 90 commonly enriched KEGG pathways (Fig. 9*B*). Selected pathways are shown in (Fig. 9*C*), while the rest of the enriched pathways are depicted in (Supplemental Fig. S9). The findings included, *e.g.*, pathways related to the metabolism of nucleotides, glutathione, and lipids (Supp3-List of Pathways). The “Environmental Information Processing” KEGG category included pathway regulation in, *e.g.,* “cell communication,” “adhesion,” and “intracellular regulation.” Changes in the regulation of synaptic/neuronal remodeling and plasticity were suggested by the enrichment of several pathways such as regulation of actin cytoskeleton, axon guidance, and focal adhesion. The KEGG “organismal systems” category showed involvement of various neurotransmitter and hormone signaling systems in the RP pathophysiology, such as estrogen, insulin, thyroid hormone, prolactin, oxytocin, growth hormone, neurotrophins, cholinergic, glutamate, GABA, adrenergic, and dopamine pathways. In the KEGG “human diseases” category, several cancer-related pathways were also enriched, indicating common signaling phenomena with retinal neurodegeneration.

**Fig. 10:**
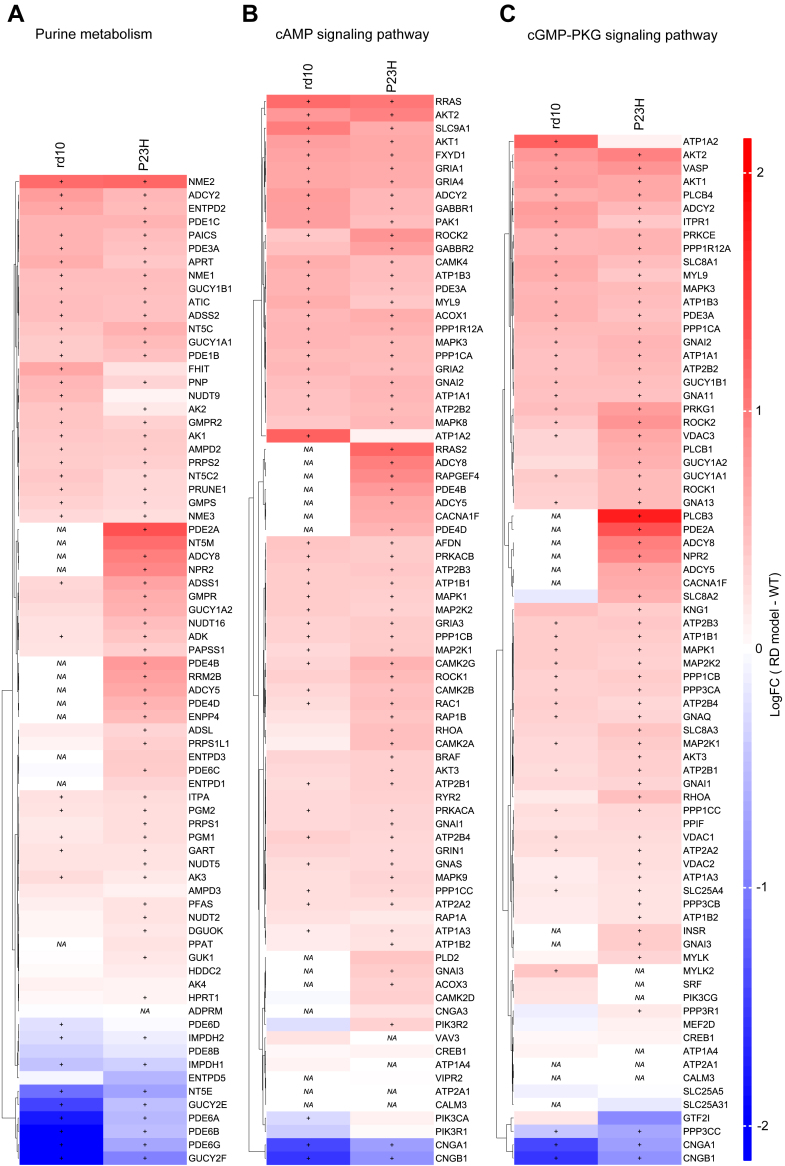
**Altered purine metabolism and cAMP/cGMP signaling pathways are robust features of the RP mouse retinal proteome.** Heatmaps show changes in the expression of the components involved in the purine metabolism pathway (*A*), cAMP signaling pathway (*B* and *C*) cGMP-PKG signaling pathway in the retinas of rd10 mice (rd10, n = 4; WT, n = 4) and P23H mice (P23H, n = 10; WT, n = 5), as compared to those of their respective WT controls. The statistical analysis was performed by the LIMMA test followed by FDR multiple testing corrections. DEPs were identified using the following criteria: *q-value* < 0.05 and log2FC > 0.5. The + signs indicate statistically significant changes from WT values. Some of the components in these pathways were not acquired (*NA*) for analysis due to missing values.

From these numerous common KEGG pathways “Purine metabolism,” “cAMP signaling pathway,” and “cGMP-PKG signaling pathway” are particularly relevant targets to therapeutic interventions (Y. (11, 14, 47, 48); therefore, they are considered here in more detail. Except for rod-enriched components that are downregulated as a direct result of rod degeneration (*e.g.*, PDE6A, PDE6B, PDE6G, GUCY2E, GUCY2F, NT5E, IMPDH1), the purine metabolism pathway showed an overall trend towards upregulation in both rd10 and P23H retinas (Fig. 10*A*). IMPDH1, which catalyzes the synthesis of xanthine monophosphate from inosine-5′-monophosphate is downregulated, whereas ADSS1, an enzyme that plays a role in the conversion of inosine monophosphate to adenosine monophosphate is upregulated in RP retinas. However, IMPDH1 is enriched in photoreceptors so it could be decreased simply because of degeneration (44). Several subtypes of adenylyl cyclases (ADCY2, ADCY5, and ADCY8), which catalyze the formation of cAMP from ATP, were significantly upregulated in P23H retinas. In addition, several cAMP- and cGMP-degrading phosphodiesterases (PDE1C, PDE3A, PDE4B, PDE4D) were upregulated in P23H retinas. *Adcy2* and *Adcy8* mRNA primarily localizes to mouse cone bipolar cells (CBCs), whereas *Adcy5* mRNA is expressed most abundantly in amacrine cells (AC) and retinal ganglion cells (RGCs) (44) (Supplemental Fig. S10). *Pde4b* is mostly expressed in CBCs while *Pde4d* expression is rather low but may be primarily expressed in astrocytes (44). ADCY2, PDE1C, and PDE3A proteins are overexpressed in both rd10 and P23H mouse retinas. *Adcy2* mRNA is detected most highly in CBCs, horizontal cells (HCs), and RGCs, *Pde1c* appears to localize primarily to CBCs, and *Pde3a* to HCs and Acs in the mouse retinas (44).

A similar trend toward upregulation as in the “purine metabolism” pathway is seen in the cAMP- and cGMP-signaling pathways; in contrast, the rod-enriched cyclic nucleotide-gated channels alpha and beta 1 (CNGA1, CNGB1) are highly downregulated (Fig. 10, *B* and *C*). Both homologs of rho kinases (ROCK1 & ROCK2) are upregulated in RP retinas. The increased activity of the rho kinases has been implicated frequently in neurodegeneration (49). Calcium/calmodulin-dependent protein kinases CAMK2G and CAMK4 are also upregulated in rd10 and P23H retinas. *Camk2g* and *Camk4* mRNAs are mostly localized to ACs and RGCs, or CBCs, respectively (44). Overall, it appears that the inner retina responds to photoreceptor degeneration by upregulating proteins that belong to the purine metabolism and cAMP/cGMP pathways, suggesting increased activity of the cAMP and/or cGMP second messengers in retinal compartments that are not affected primarily.

**Fig. 11:**
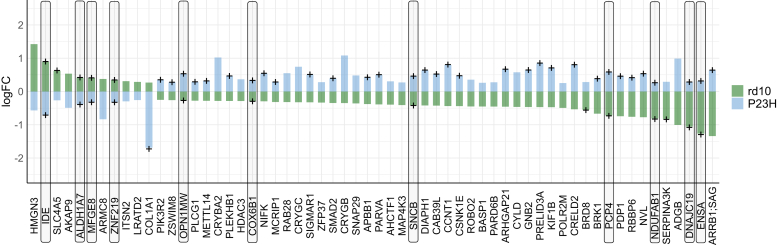
**A small number of proteins show changes in expression in opposite directions in the rd10 and P23H mouse retinas.** The bar chart shows proteins with opposite-direction changes of expression in the retinas of rd10 mice (rd10, n = 4; WT, n = 4) and P23H mice (P23H, n = 10; WT, n = 5). The criterion for identifying these proteins was that the direction of change in expression was opposite, and the absolute quantitative difference was log2FC > 0.5 between the rd10 and P23H mouse retinas. On the y-axis, log2FC is shown as the difference between rd10 or P23H *versus* their respective WT controls. Statistical analysis was performed with the LIMMA test followed by FDR multiple testing corrections. The + signs indicate statistically significant changes: *q-value* < 0.05. Ten of the DEPs for which the changes in opposing directions for the two RP models are both statistically significant are highlighted with *boxes*.

The retinal proteomes of both rd10 and P23H mice generally exhibited changes in the same direction, either upregulated or downregulated, compared to their respective WT controls; however, the extent of these changes varied with the progression of the disease (see example: Supplememental Figs. S6–S8). However, some proteins exhibited opposite expression patterns (absolute log2FC > 0.5) (Fig. 11), with only 10 of these protein changes (ALDH1A7, COX6B1, DNAJC19, ENSA, IDE, MFGE8, NDUFAB1, OPN1MW, PCP4, ZNF219) being statistically significant in both mouse models (*q-value* < 0.05). Alpha-endosulfine (ENSA) was downregulated and insulin-degrading enzyme (IDE) was upregulated in rd10 mouse retinas, while the opposite was observed in P23H mouse retinas; these proteins are associated with insulin catabolism and secretion (50). DEPs or enriched pathways that are only detected in rd10 retinas, or only in P23H retinas are reported in the supplementary data (Supp2 - List of Proteins and Supp3 - List of Pathways).

## Discussion

This study provides a comparative analysis of retinal proteomes from three distinct IRD mouse models. Two of the models represent RP, while one model is more closely associated with LCA2. Mice were housed, and retinal samples were collected in two different institutions, while LC-MS/MS was performed in three different laboratories. However, for peptide search and statistical analysis, the LC-MS/MS data was aggregated, and data analysis was performed simultaneously by the same researcher following the same parameters for each dataset. The data provides accurate relative proteome quantification across the three different IRD models. The data have been meticulously analyzed and curated, offering a valuable resource to identify distinct and reproducible proteomic changes occurring among these three IRD models, and in particular, between the RP models. Indeed, our analysis reveals highly convergent retinal proteomic changes within the RP models (rd10 and P23H), whereas the LCA2 (*Rpe65*^−/−^) samples show several uniquely altered proteomic pathways.

### Overall Characteristics of the Disease Models and Proteomics Data

Rod-enriched markers and scotopic ERG responses were significantly dampened in all three models, confirming rod degeneration (Fig. 2*A*). However, the pathologic characteristics of the *Rpe65*^−/−^ mice were distinct. The anatomic degeneration of rods is minor at this disease stage in the *Rpe65*^−/−^ mice, but the rod-mediated function is impaired due to full blockade of the RPE-mediated visual cycle in these animals (24). The cone photoreceptors quickly die in *Rpe65*^−/−^ mice, and from birth, they fail to respond to light (25). Because only 3% of mouse photoreceptors are cones (51), and their distribution across the retina is rather homogenous (46), their loss does not cause a clear change in the retinal macrostructure.

In rd10 mice the progression of anatomic rod degeneration is fast, and in P23H mice it occurs at an intermediate rate. Cone degeneration is a secondary outcome and occurs much later in both models (Fig. 2*A*) (18, 23). These characteristics of the different models are important to bear in mind when drawing conclusions from the data presented in this work. We expect that in the retinas of *Rpe65*^−/−^ mice, proteomic changes are due to loss of photoreceptor function, and less so from anatomic degeneration. In both RP models, the photoreceptors, particularly cones, still signal well to the downstream neurons in the inner retina, and we interpret the proteomics changes in their retinas as being caused more by anatomic rod degeneration than in *Rpe65*^−/−^ mice. Nevertheless, markers of inflammation and cell stress state were relatively similarly upregulated in all three models (Fig. 2*C*).

Since our DDA datasets did not detect many differences between the rd10 and P23H models, we performed additional analyses in the DIA mode. In general, the DIA mode generated over two-fold proteome coverage than did the DDA mode: 5913 vs 2688 in rd10, and 6843 vs 3150 in P23H (Supplemental Fig. S2). A total of 257 proteins were exclusively identified using the DDA mode in both rd10 and P23H models, whereas approximately 4251 proteins were uniquely detected through the DIA analysis mode. Despite the retinal samples being collected in two different laboratories, the relative quantification accuracy compared to their respective WT controls was similar for the two analysis modes, as illustrated by the comparison of retinal marker proteins (Supplemental Fig. S4).

### Common Modes of Regulation in the Retinal Proteomes of the Three IRD Models

Only 28 proteins showed significant regulation commonly between all the rd10, P23H and *Rpe65*^*−/−*^ models according to our statistical threshold. Three known RD-associated proteins GNB1 (52), LMPDH1 (53), and ARL3 (54) were downregulated. Common upregulation was observed for proteins that respond to inflammation and cell stress (APOE, ATP6V1G2, CLU, CORO1C, GFAP, LASP1, LMNA, LRP1, MSN, NRCAM, and TWF1), vascular remodeling and proliferation (CTNNB1, GAP43, TF, VCL, VIM), oxidative stress (GSTT1), or apoptosis (CAPN2, NEBL, NME2). The classic inflammatory markers APOE, GFAP, and VIM indicated stress-phenotype severity in the following order: rd10 > *Rpe65*^*−/−*^ > P23H.

Five DEPs showed opposite regulation in the retina of the LCA model as compared to both RP models. Thus, DCLK1, ARHGAP1, and TPPP3 showed downregulation in *Rpe65*^*−/−*^ samples but were upregulated in rd10 and P23H. DCLK1 is a doublecortin-like kinase that regulates microtubule binding (55), and it has been shown to promote neuronal survival, growth cone formation, and axon regeneration in retinal ganglion cells (RGCs) after axotomy (56). ARHGAP1 belongs to the family of Rho GTPase-activating proteins that play a role in axon guidance (57). TPPP3 belongs to the family of tubulin polymerization-promoting proteins, and its upregulation may promote axon regeneration (58). These findings are consistent with the interpretation that retinas affected with primary rod degeneration (rd10 and P23H) would display more remodeling than retinas with little anatomic rod degeneration (*Rpe65*^*−/−*^).

HMGB2 and PDC were upregulated in *Rpe65*^*−/−*^ but downregulated in rd10 and P23H mouse retinas. HMGB2 is a pro-inflammatory protein that can cause photoreceptor death when released into the extracellular space upon oxidative stress via the activation of NF-κB/NLRP3 signaling pathways (Y (59). *Vice versa*, the knockdown of HMGB2 suppresses cell death in a light-induced retinal degeneration (LIRD) mouse model (44). HMGB2 is a relatively rod-enriched protein, which could explain its downregulation in the rd10 and P23H mouse models. PDC, phosducin, is a phosphoprotein expressed in the rod inner and outer segments; it participates in regulating the light sensitivity of synaptic transmission between photoreceptors and ON-CBCs (60). The specific role of PDC is not fully understood but it is hypothesized to be crucial for rod photoreceptor adaptation to bright light (61). PDC is decreased in both RP models, particularly strongly in rd10 retinas, likely due to the decrease in volume of the rod outer segment. The slight upregulation of PDC protein in *Rpe65*^*/-*^ retinas is a more intriguing finding. It is notable that the *Rpe65*^*−/−*^ mice retain some rod-mediated ERG responses and residual visual function (43) even though their chromophore content enabling phototransduction is undetectable, at least with typical HPLC-based methods (20). It remains enigmatic what enables the rods of *Rpe65*^*−/−*^ mice to function without the classical visual cycle, but it appears that their rod system is supersensitized. One interesting hypothesis is that some compensatory mechanisms, perhaps including PDC, scale up rod-mediated signaling to counteract visual cycle dysfunction (62). Based on published single-cell RNA-sequencing data (63), *Pdc* gene expression is increased in *Rpe65*^−/−^ mouse rod cells but lowered in bipolar cells and Müller glia cells compared to corresponding WT cells.

The commonly regulated 28 DEPs are associated with four KEGG pathways. Downregulation of the *phototransduction* pathway is expected in all models; however, it varies between the models and follows the severity of rod death rather than phototransduction efficiency (Supplemental Fig. S6). The pathways *bacterial invasion of epithelial cells* and *tight junction* mostly correspond to the regulation of cytoskeleton-related proteins such as myosins, alpha-actinins, ezrin, tight junction protein ZO-1, and alpha-tubulins, which play a role in functions such as maintenance of cell morphology, cell motility, and dynamic stability of the cytoskeleton. We assume that their upregulation is a survival mechanism aimed at counteracting degeneration (64). Gamma-aminobutyric acid (GABA) is the primary inhibitory neurotransmitter in our nervous system, including in the retina (65, 66). Based on our data, the GABA pathway is modulated in IRD retinas regardless of disease mechanism. However, it is far more robustly regulated in the retinas of rd10 mice, and particularly P23H mice, compared to *Rpe65*^*−/−*^ mice (Supplemental Fig. S7). Regulated components in the *GABAergic synapse* pathway include, *e.g.*, vesicular inhibitory amino acid transporters and sodium- and chloride-dependent GABA transporters. The primary enzyme responsible for catalyzing the production of GABA from glutamate, GAD1, is significantly upregulated in rd10 and P23H retinas. These data are in line with literature suggesting catabolism of GABA and related metabolites as a hallmark of retinal degeneration (62, 67).

### The *Rpe65*^*−/−*^ Model Displays Several Proteomic Features that are Distinct From the RP Models

Metabolic imbalance has been demonstrated as an early pathological event in RD including *Pde6* mutation-associated diseases (58), and candidate therapies are being investigated to address the problem (68, 69). Our analysis from adult mouse retinas demonstrates significant regulation of several proteins involved in the mitochondrial electron transport chain; e.g., oxidative phosphorylation (OXPHOS) and tricarboxylic acid (TCA) cycle in the *Rpe65*^*−/−*^ mice, but less so in the RP models. Based on this analysis, altered mitochondrial function in adult *Rpe65*^−/−^ mouse retinas seems evident, whereas the retinas in adult rd10 and P23H mice may be better able to maintain mitochondrial function via homeostasis. Several findings distinguish the LCA2 model from the two RP models. For example, fatty acid-binding protein 5 (FABP5) is downregulated 2.6-fold in *Rpe65*^−/−^ retinas but upregulated 0.4-fold in rd10 and P23H retinas. Many OXPHOS or TCA cycle components that have normal expression in rd10 and P23H retinas show significant alterations from WT levels in *Rpe65*^−/−^ mouse retinas (Fig. 7*A* and Supplemental Fig. S8). The opposing regulation of COX5A and COX5B in *Rpe65*^−/−^ retinas is particularly noteworthy as the upregulation of COX5B and downregulation of COX5A could indicate cellular stress under anaerobic conditions (70). Additionally, the upregulation of mitochondrial aconitate hydratase (ACO2) and downregulation of cytosolic aconitate hydratase (ACO1) represent distinct metabolic features in the *Rpe65*^−/−^ retinas.

In addition to OXPHOS- and TCA cycle-related changes, another distinct feature of the *Rpe65*^*−/−*^ model includes robust downregulation of multiple lens crystalline proteins (Fig. 4). The lens crystalline proteins are believed to play an important protective role in RGC survival and regeneration (71, 72). While tens of crystalline proteins were downregulated monotonically in the retinas of *Rpe65*^−/−^ mice, many of the same proteins showed increased mean expression (not necessarily significant) in the retinas of rd10 and particularly P23H mice (Fig. 4). Apart from cell metabolism, several pathways related to DNA (cell cycle) and RNA (spliceosome) functions, protein translation (Aminoacyl-tRNA biosynthesis), as well as neurodegenerative diseases, were also distinctly regulated in the *Rpe65*^*−/−*^ mouse retinas (Figs. 6 and 7*B*). The overexpression of many histone and nucleosome-related proteins in the *Rpe65*^*−/−*^ retina suggests altered chromatin accessibility (Fig. 5*A*). This observation implies a potential epigenetic regulation mechanism affecting gene expression and cellular functions. These phenomena were not observed in either of the RP-model retinas, which is somewhat surprising. Decreased chromatin accessibility has been identified as a characteristic feature in retinal diseases, mostly in age-related macular degeneration (73), and HDAC11 in particular has been proposed as a therapeutic target. Further investigation of this epigenetic aspect in RD is warranted.

### Commonly Regulated Retinal Proteome in the rd10 and P23H Models of RP

Hundreds of proteins and tens of KEGG pathways were found to be co-regulated in rd10 and P23H mouse retinas (Fig. 9 and Supplemental Fig. S9), indicating that the two different RP models possess convergent downstream pathophysiological pathways, regardless of their primary mechanisms of rod degeneration. Identification of these common pathophysiological pathways is advantageous for the conceptualization of novel DMTs for RP. Accordingly, all of the proteomic data from this study is carefully curated and made publicly available (See Data Availability). Here, we highlight some of the common pathways that we believe could be directly relevant with respect to therapeutic strategies. Notably, pathways under the “cellular processes” KEGG category (*e.g.*, apoptosis, autophagy, regulation of actin cytoskeleton) contained the largest number of DEPs identified in our study. This result is likely a direct consequence of cellular stress response. Another example is the KEGG category “organismal systems,” which includes many signaling pathways comprising hundreds of potentially targetable G protein-coupled receptors (GPCRs) and related proteins.

Regulation of the actin cytoskeleton is a crucial function in injury-induced axon retraction by rod cells (74). The overall upregulation of several actin and myosin regulation proteins in the retinal proteome of the RP models indicates retinal remodeling. Also, the rho-associated protein kinase (ROCK) pathway, FGF2-HRAS pathway, and RhoA-LIMK-Cofilin pathway were found to be enriched in our analysis that requires further investigation regarding their roles in actin dynamics and retinal remodeling. Also noteworthy, the *Proteoglycans in cancer* pathway are enriched in the RP retinal proteome, with significant upregulation of proteins related to chondroitin sulfate, heparan sulfate, and hyaluronan. Such proteoglycans regulate axon guidance and synapse formation during the development of the nervous tissue (75), and thus their upregulation could play a role in adaptive plasticity. On the other hand, proteoglycan-deficient retinas display a retinal-degeneration phenotype (76). Significant enrichment of phosphatidylinositol signaling-related proteins is also observed in the RP retinal proteome, primarily attributable to the marked upregulation of phosphatidylinositol C phospholipases. These enzymes play a crucial role in the metabolism of phosphatidylinositol 4,5-bisphosphate into second messengers such as inositol (1,4,5) trisphosphate and diacylglycerol, besides the regulation of lipid-signaling pathways in a calcium-dependent manner (77). Dysfunctional phosphatidylinositol metabolism can cause retinal degeneration (78, 79). The observed upregulation of this pathway in the RP models may indicate calcium dysregulation, a hallmark of neurodegeneration (80, 81). Further investigation of these changes is warranted to better understand their underlying mechanisms and to identify potential therapeutic targets for RP. In addition, the increased expression of glutathione S-transferases and peroxiredoxin-6 (PRDX6) may represent a defense mechanism to counteract oxidative stress, which represents an attractive target to mitigate secondary cone death in RP (82).

The *Purine metabolism* pathway in the retina is distinctly enriched in both RP models as well as the associated cAMP signaling and cGMP-PKG signaling pathways. Many proteins included in these pathways are highly rod-enriched and consequently downregulated (*e.g*., PDEs & CNGA1/B1). Membrane-bound guanylate cyclases (*e.g.*, GUCY2E and GUCY2F), which are also rod-enriched (44), are downregulated, whereas soluble guanylate cyclases (sGC) (*e.g.*, GUCY1a1, GUCY1a2, and GUCY1b1), which are not photoreceptor-enriched (44), are upregulated. This regulation pattern indicates that sGC may secondarily modulate the levels of cGMP during rod degeneration. The changes in the expression of guanylate cyclases are in line with the implicated important role of cGMP dysregulation during RD (83). Moreover, our data demonstrated the upregulation of several adenylate cyclases (ADCYs) and their downstream targets (*e.g*., EPAC2, MAPK8, and RRAS2) in RP retinas, with no detected downregulations in these components. Possibly as a response to increased ADCY activity, many components of the calcium/calmodulin-dependent protein kinases are upregulated.

Pharmacologically targeting the dysregulation of intracellular signaling molecules such as cAMP and cGMP offers a potential DMT strategy to treat retinal degeneration (48, 84). The second messengers cAMP and cGMP are regulated by ADCY and GUCY enzymes which synthesize them, and phosphodiesterases which degrade them, to maintain a balanced level. The role of the cGMP-PKG pathway in retinal degeneration has been extensively studied (85, 86, 87), and pharmacological inhibition of excessive cGMP activity protects the retinas in rd1, rd2, and rd10 RP mouse models (48). It remains to be determined if suppression of cAMP activity could also be protective in RP, but indirect evidence from studies using GPCR drugs to suppress cAMP production suggests therapeutic potential (88, 89, 90). Another intriguing question is if simultaneous cAMP and cGMP inhibition could provide additive or even synergistic therapeutic effects.

### Methodological Limitations and Future Perspectives

In proteomic analyses of bulk retinal tissues, such as those undertaken in this study, there is a concern regarding the unequal volumes of retinal layers, particularly when comparing samples from healthy *versus* degenerative states. Given that rods constitute 97% of the outer retinal cells in mice, the proportion of outer retina volume in the homogenate used for proteomic analysis is decreased in the RD samples compared to those from WT mice. This disparity results in an apparent decrease in proteins enriched in rods. *Vice versa*, the proteins in surviving retinal-cell populations (*e.g.*, inner retina compartment) may seem proportionally upregulated, even if their expression per cell remains unchanged. Our datasets revealed a higher number of overexpressed proteins, suggesting alterations in protein expression not solely attributable to a decrease in rod volume, but possibly to the increased proportional volume of the inner retinal compartment in RD samples. Therefore, findings from bulk retina proteomics (and other omics approaches) in the RD context need to be interpreted with caution, and firm conclusions should not be drawn from stand-alone data. However, the primary findings presented in this manuscript, such as changes in cyclic nucleotide metabolism in RP models and alterations in OXPHOS, spliceosome, and TCA cycle in the LCA2 model, are so clearly supported by the data, and in the case of RP, reinforced by existing literature, that we believe they reflect genuine signaling changes rather than misinterpretations due to changes in retinal volume.

While awaiting the widespread application and cost-reduction in single-cell proteomics, immunohistochemistry can offer insights into localized changes in protein expression, albeit with limited quantification potential. Also, some methods to fractionate different layers of the retina have been developed (91). Alternatively, specific cell populations can be isolated using techniques such as fluorescence-activated cell sorting (FACS) prior to LC-MS/MS analysis.

## Conclusion

Mutation-agnostic disease-modifying therapies for the various forms of retinitis pigmentosa are feasible due to shared pathological phenomena downstream from primary rod degeneration. Here, we highlighted some potential therapeutic targets, particularly the cyclic-nucleotide second-messenger pathways, that are practicable not only for novel drugs but also for drug-repurposing strategies. This study contributes valuable data to the public research domain, supporting the pursuit of the first broadly applicable therapeutics for RP.

## Data Availability

The mass spectrometry proteomics data have been deposited to the ProteomeXchange Consortium (92) *via* the PRIDE partner repository (93) with the dataset identifier PXD052547 (UCI_DDA_P23H_cohort 1), PXD052549 (UCI_DDA_rd10_DR_cohort 2), PXD052554 (UCI_DDA_*Rpe65*^−/−^ cohort 1), PXD052555 (UCI_DDA_rd10_CLR_cohort 1) or *via* skyline and Panorama public repository (94) with the dataset identifiers PXD052698 (UEF_DIA_rd10_DR_cohort 3) and PXD052598 (UEF_DIA_P23H_cohort 2). Examples of how to access fully annotated spectra of the deposited data are illustrated in supp1emental Figs. S11–S14.

## Supplemental data

This article contains supplemental data (Supp1: supplementary figures, Supp2: supplementary list of proteins, Supp3: supplementary list of pathways, and Supp4: sample characteristics).

## Conflict of interest

K. P. is a consultant for Polgenix Inc. and serves on the Scientific Advisory Board at Hyperion Eye Ltd. D.F. is co-founder of MedBiome Inc, a company developing precision microbiome therapeutics and nutrition. All other authors declare that they have no conflicts of interest with the contents of this article.

## Supplementary Data

Supp1-Figures_no_figures

Supp2-Proteins_list

Supp3-Pathways_list

Supp4-Sample_characteristics
