# Supplementary material for: Retinal Proteome Profiling of Inherited Retinal Degeneration Across Three Different Mouse Models Suggests Common Drug Targets in Retinitis Pigmentosa
Source: Mol Cell Proteomics. 2024 Oct 9;23(11):100855. doi: 10.1016/j.mcpro.2024.100855 (PMC11602984; doi:10.1016/j.mcpro.2024.100855)
Supplement: Supp1-Figures_no_figures [file mmc1.docx]

# **SUPPLEMENTARY DATA (Supp1-Figures)**

**Retinal proteome profiling of inherited retinal degeneration across three different mouse models suggests common drug targets in retinitis pigmentosa**

Ahmed B. Montaser^1*^, Fangyuan Gao^2^, Danielle Peters^3^, Katri Vainionpää^1^, Ning Zhibin^3^, Dorota Skowronska-Krawczyk^2^, Daniel Figeys^4^, Krzysztof Palczewski^2^, Henri Leinonen^1*^.

^1^School of Pharmacy, Faculty of Health Sciences, University of Eastern Finland, P.O. Box 1627, FI-70211 Kuopio, Finland

^2^Center for Translational Vision Research, Department of Ophthalmology, Gavin Herbert Eye Institute, University of California, Irvine, Irvine, CA, 92697, USA; Department of Physiology and Biophysics, Department of Chemistry, Department of Molecular Biology and Biochemistry; University of California, Irvine, Irvine, CA, 92697, USA

^3^Ottawa Institute of Systems Biology, University of Ottawa, Ottawa, ON, Canada

^4^Department of Biochemistry, Microbiology and Immunology, University of Ottawa, Ottawa, ON, Canada

^*^ Corresponding authors: [ahmed.montaser@uef.fi](mailto:ahmed.montaser@uef.fi); [henri.leinonen@uef.fi](mailto:henri.leinonen@uef.fi)

Contents

[**SUPPLEMENTARY DATA (Supp1-Figures)** 1](#_Toc176427108)

[1. DDA dataset protein identifications overview 3](#_Toc176427109)

[2. DIA versus DDA dataset, protein-identification overview 4](#_Toc176427110)

[3. Genes expression of retinal markers used in this study 5](#_Toc176427111)

[4. DIA versus DDA datasets protein-quantification comparison 6](#_Toc176427112)

[5. Comparison of DEPs in the (DDA_Rd10_DR) and (DDA_Rd10_CLR) datasets 7](#_Toc176427113)

[6. Heatmap of selected common mouse KEGG pathways in the three RD mouse models 8](#_Toc176427114)

[7. Common enriched KEGG pathways in rd10 and P23H mouse models 10](#_Toc176427115)

[8. Gene expression of Adcys in the different cell types of the mouse eye. 11](#_Toc176427116)

[9. Access to fully annotated spectra. 11](#_Toc176427117)

### DDA dataset protein identifications overview

**Supplementary Figure 1.** Venn diagram showing the total number of proteins identified in the DDA datasets for retinas of rd10 (DR, dark reared; CLR, cyclic-light reared), P23H, and Rpe65^-/-^ mice.

### DIA versus DDA dataset, protein-identification overview

**Supplementary Figure 2.** Venn diagram showing the total number of proteins identified in the DDA and DIA datasets for retinas of rd10_DR, rd10_CLR, P23H, and Rpe65^-/-^ mice.

### Genes expression of retinal markers used in this study

**Supplementary Figure 3**. Mouse eye gene expression dot plot showing retinal gene markers (downloaded from Cellxgene online tool; https://cellxgene.cziscience.com/gene-expression, accessed: 12 March 2024).

### DIA versus DDA datasets protein-quantification comparison

**Supplementary Figure 4**. Selected marker proteins, showing pattern of expression as log2FC values on the y-axis of different retinal datasets; (A) dark-reared rd10 mice analyzed in two different conditions, using the DDA method at UCI (n=6) and the DIA method at UEF (n=4); and (B) vivarium-housed P23H mice analyzed in two different conditions, using the DDA method at UCI (n=7) and the DIA method at UEF (n=10).

### Comparison of DEPs in the (DDA_Rd10_DR) and (DDA_Rd10_CLR) datasets

**Supplementary Figure 5**. Heatmap showing the DEPs of retinas from rd10 mice in two rearing conditions: cyclic light rearing (rd10_CLR), and dark rearing (rd10_DR). The double-plus signs (++) indicate statistical significance (LIMMA test with FDR correction, adjusted P value < 0.05). The single-plus (+) signs indicate statistical significance for comparisons that have some missing values (at least 2 valid values per condition done by t-test and P value < 0.01).

### Heatmap of selected common mouse KEGG pathways in the three RD mouse models

**Supplementary Figure 6**. Heatmap showing the log2FC of the detected retinal proteins that are involved in the phototransduction KEGG pathway across the three IRD mouse models. The plus signs indicate statistically significant changes (LIMMA test followed by FDR correction, adjusted P value < 0.05), while NA indicates proteins that were not quantified.

**Supplementary Figure 7**. Heatmap showing the log2FC of the detected retinal proteins that are involved in the GABAergic synapse KEGG pathway across the three IRD mouse models. The plus signs indicate statistically significant changes (LIMMA test followed by FDR correction, adjusted P value < 0.05), while NA indicates proteins that were not quantified.

**Supplementary Figure 8**. Heatmap showing the log2FC of the detected retinal proteins that are involved in the citrate cycle (TCA cycle) KEGG pathway across the three IRD mouse models. The plus signs indicate statistically significant changes (LIMMA test followed by FDR correction, adjusted P value < 0.05), while NA indicates proteins that were not quantified.

### Common enriched KEGG pathways in rd10 and P23H mouse models

**Supplementary Figure 9.** Common enriched KEGG pathways in retinas from rd10 and P23H mice, highlighting selected pathways that were described in the Results section (Figure 9) and in the Discussion of the main manuscript.

### Gene expression of Adcys in the different cell types of the mouse eye.

**Supplementary Figure 10**. Gene expression dot plot for the mouse eye, downloaded from Cellxgene online tool: (https://cellxgene.cziscience.com/gene-expression, accessed: 12 March 2024).

### Access to fully annotated spectra.

**For DDA datasets**

- In PXD052547, find UCI_DDA_P23H_cohort1.zip file.
- In PXD052549, find UCI_DDA_rd10_DR_cohort2.zip file.
- In PXD052554, find UCI_DDA_Rpe65-/- cohort1.zip file.
- In PXD052555, find UCI_DDA_rd10_CLR_cohort1.zip file.

After unzipping these files, the maxquant parameter file (mqpar) can be loaded into Maxquant software directly (as shown in supplementary Figure 11). In the Maxquant visualization tab, the full annotated spectra of all identifications can be visualized (as shown in supplementary Figure 12)

**Supplementary Figure 11.** Retrieving the whole DDA datasets into Maxquant software.

**Supplementary Figure 12.** Example of full annotated spectra of a unique peptide/protein identification.

**For DIA datasets**

Full annotated spectra can be accessed directly via Panorama Web with the provided accession numbers in the data availability section (as shown in supplementary Figures 13 & 14).

**Supplementary Figure 13.** Example of full annotated spectra of a unique peptide/protein identification via Panorama web.

**Supplementary Figure 14.** Example of full annotated spectra of a unique peptide/protein quantification via Panorama web.
