# Supplementary figures and images for: Retinal Proteome Profiling of Inherited Retinal Degeneration Across Three Different Mouse Models Suggests Common Drug Targets in Retinitis Pigmentosa
Source: Mol Cell Proteomics. 2024 Oct 9;23(11):100855. doi: 10.1016/j.mcpro.2024.100855 (PMC11602984; doi:10.1016/j.mcpro.2024.100855)

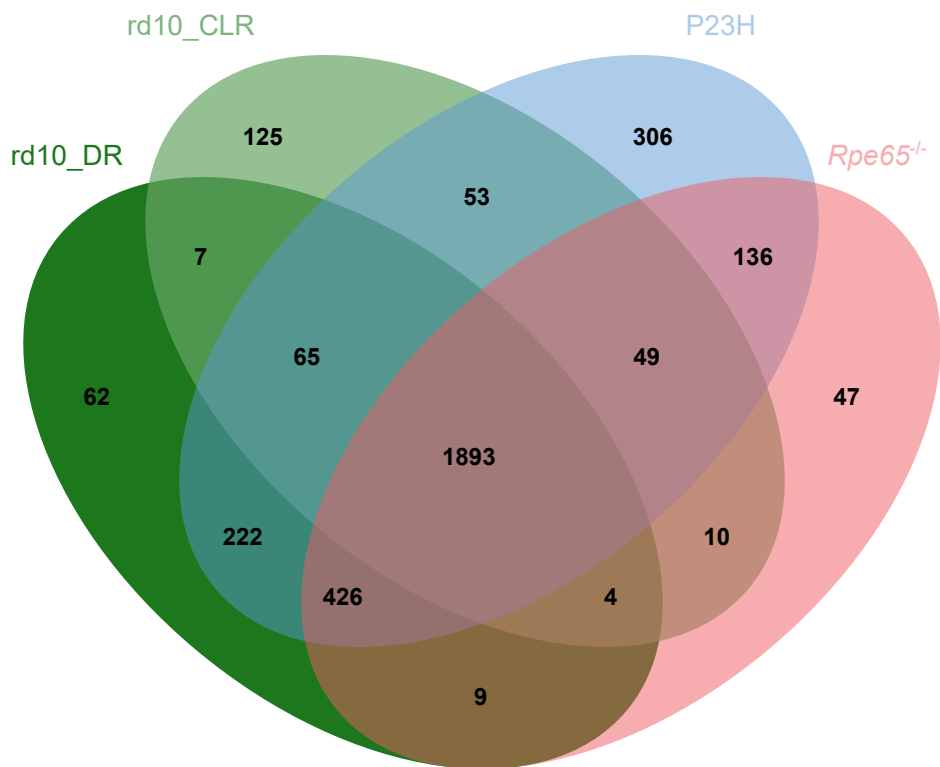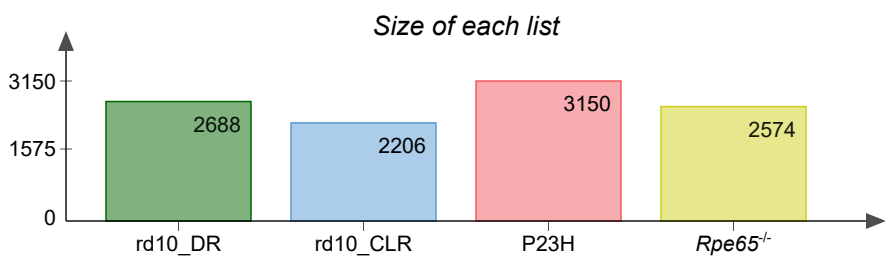

*Number of elements: specific (1) or shared by 2, 3, ... lists*

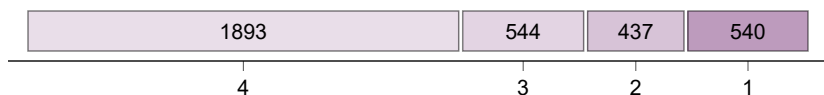

Supplement: Figure S1 [file mmc5.pdf]

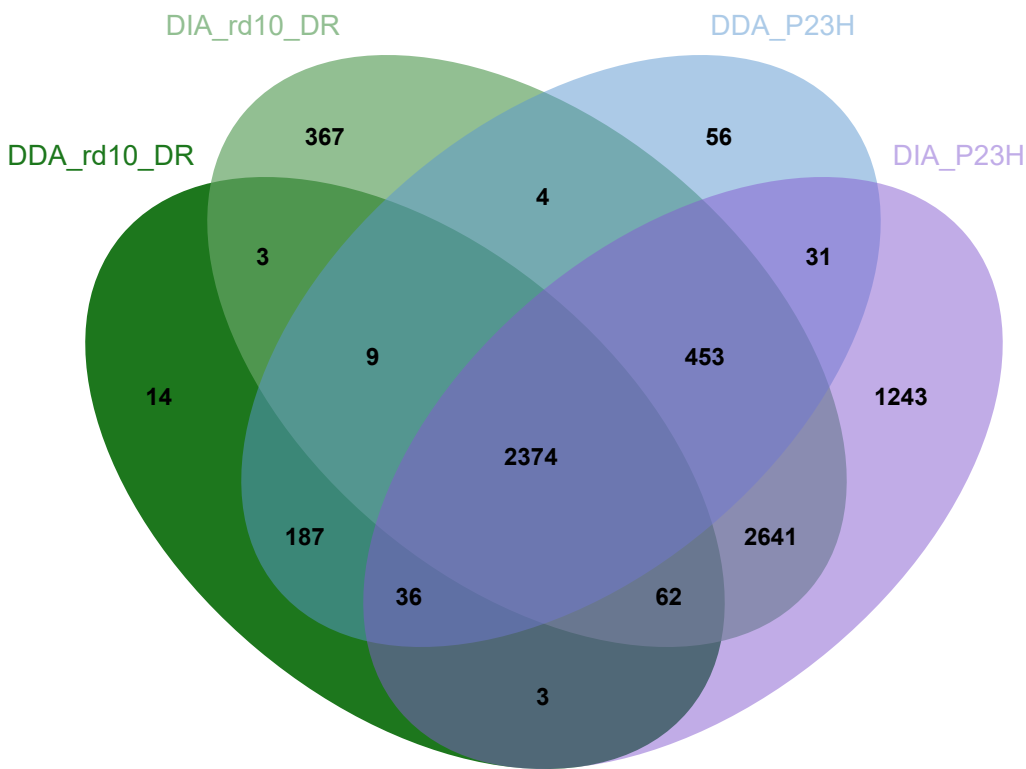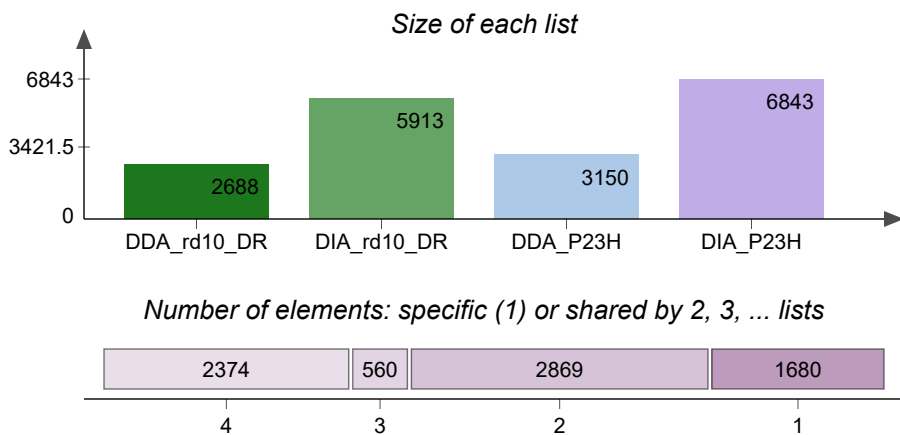

Supplement: Figure S2 [file mmc6.pdf]

Downloaded data and figures may differ in the future.

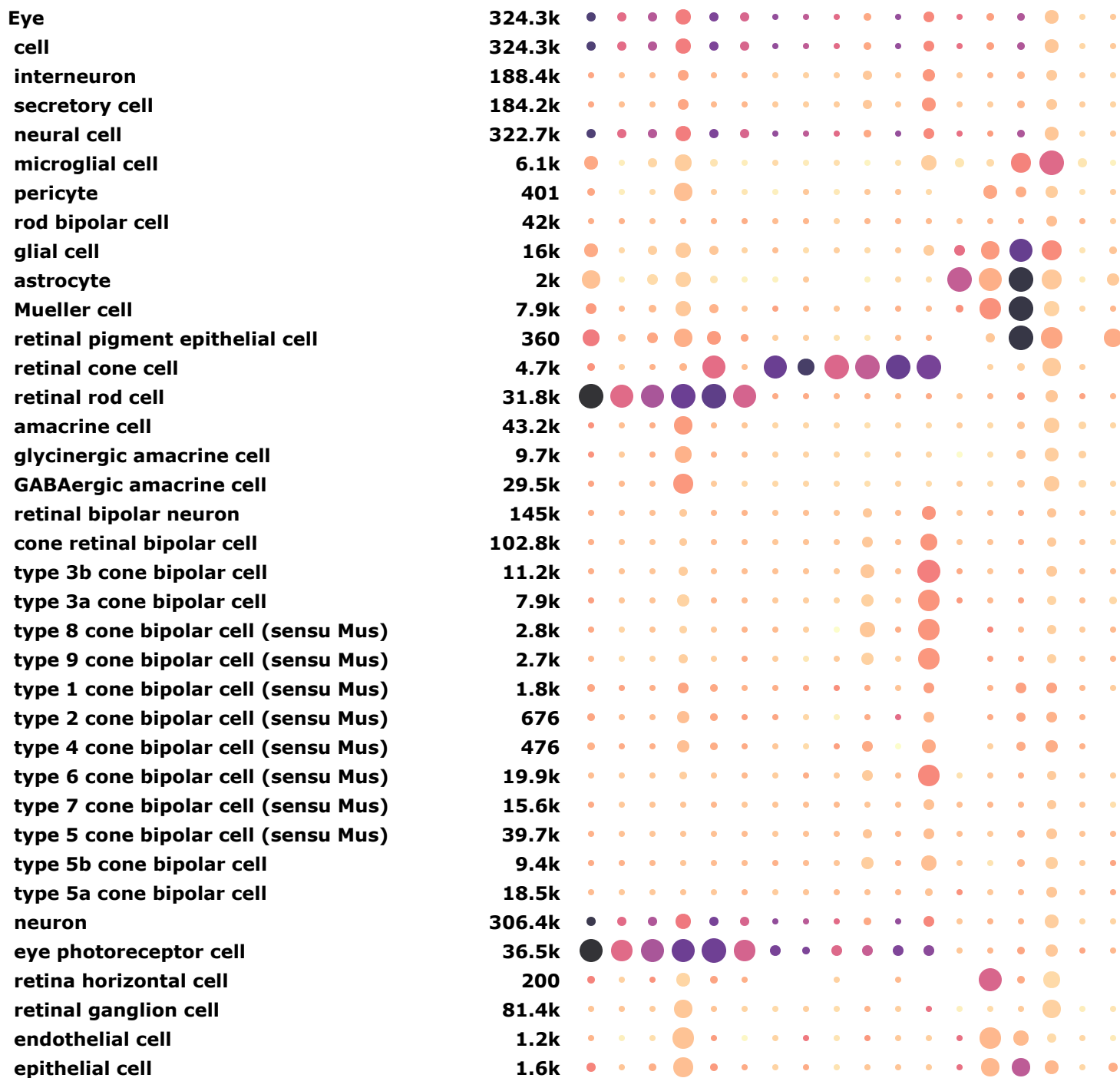

Supplement: Figure S3 [file mmc7.pdf]

A

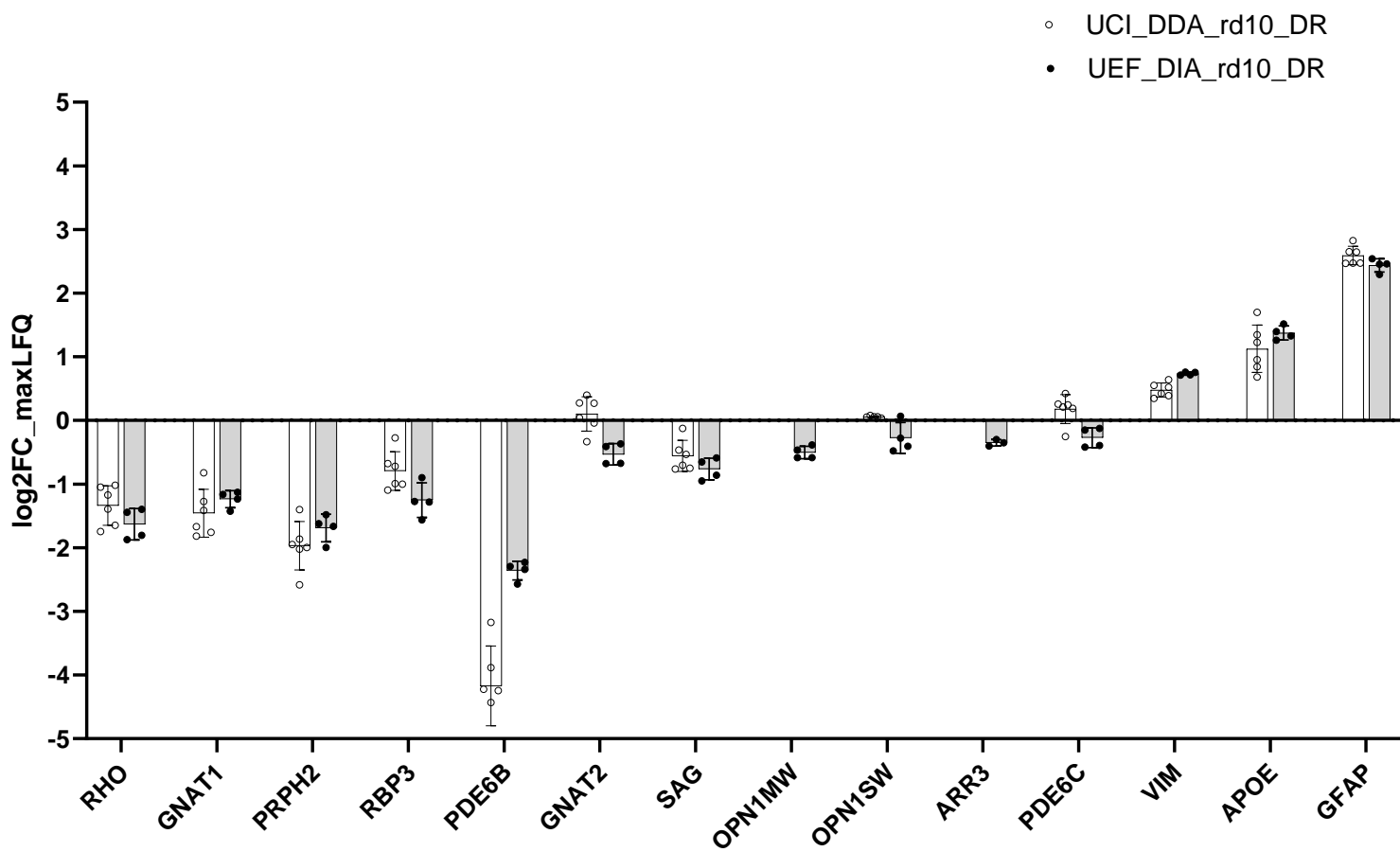

B

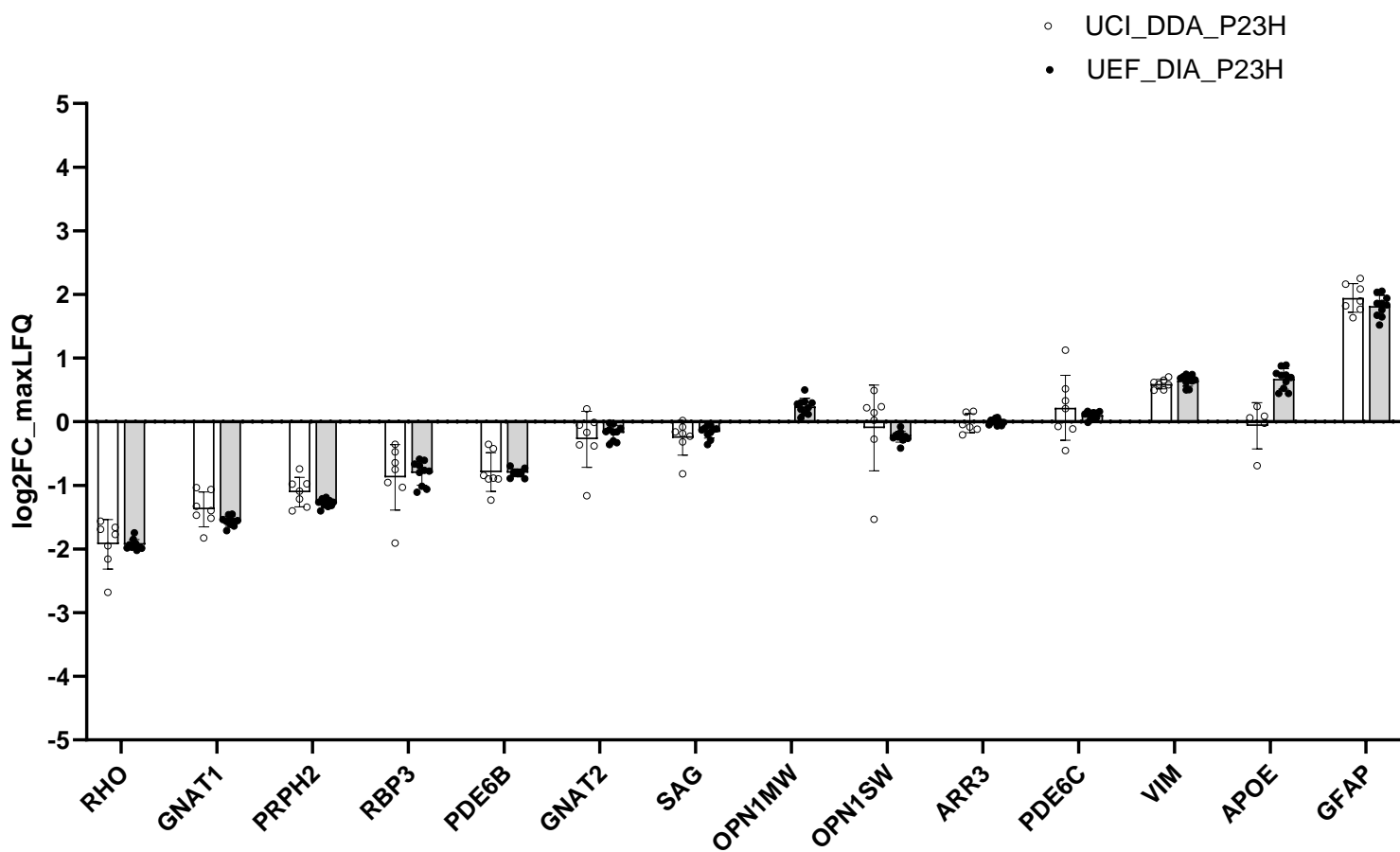

Supplement: Figure S4 [file mmc8.pdf]

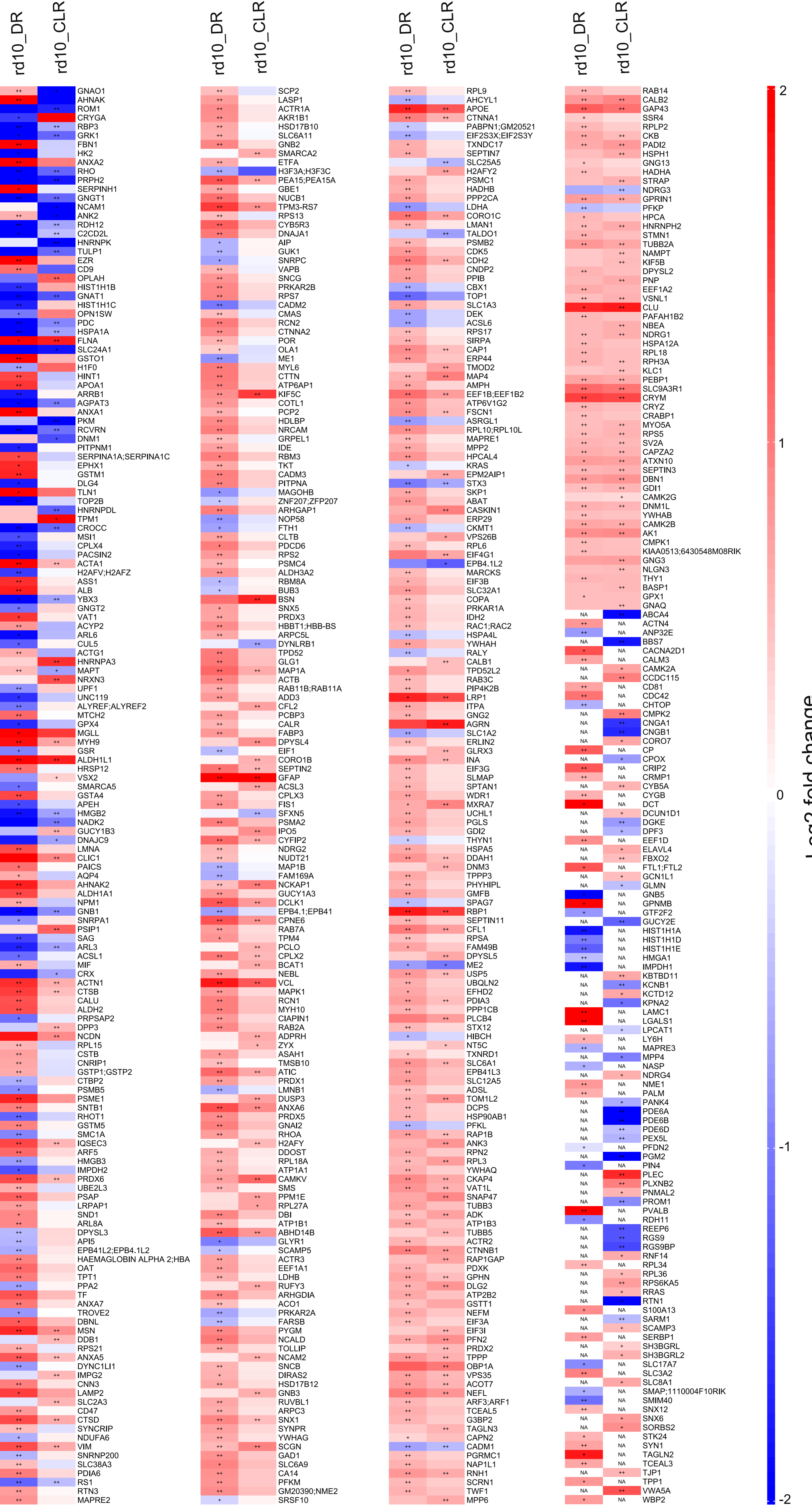

Supplement: Figure S5 [file mmc9.pdf]

# Phototransduction

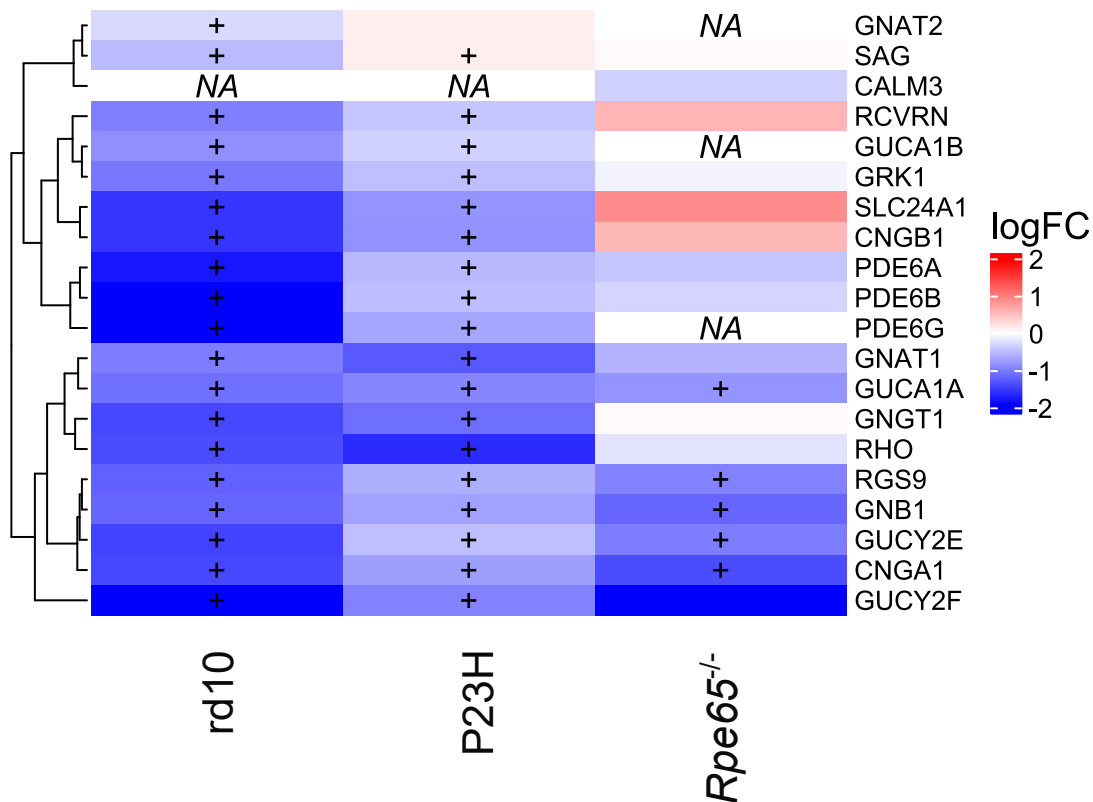

Supplement: Figure S6 [file mmc10.pdf]

# GABAergic synapse

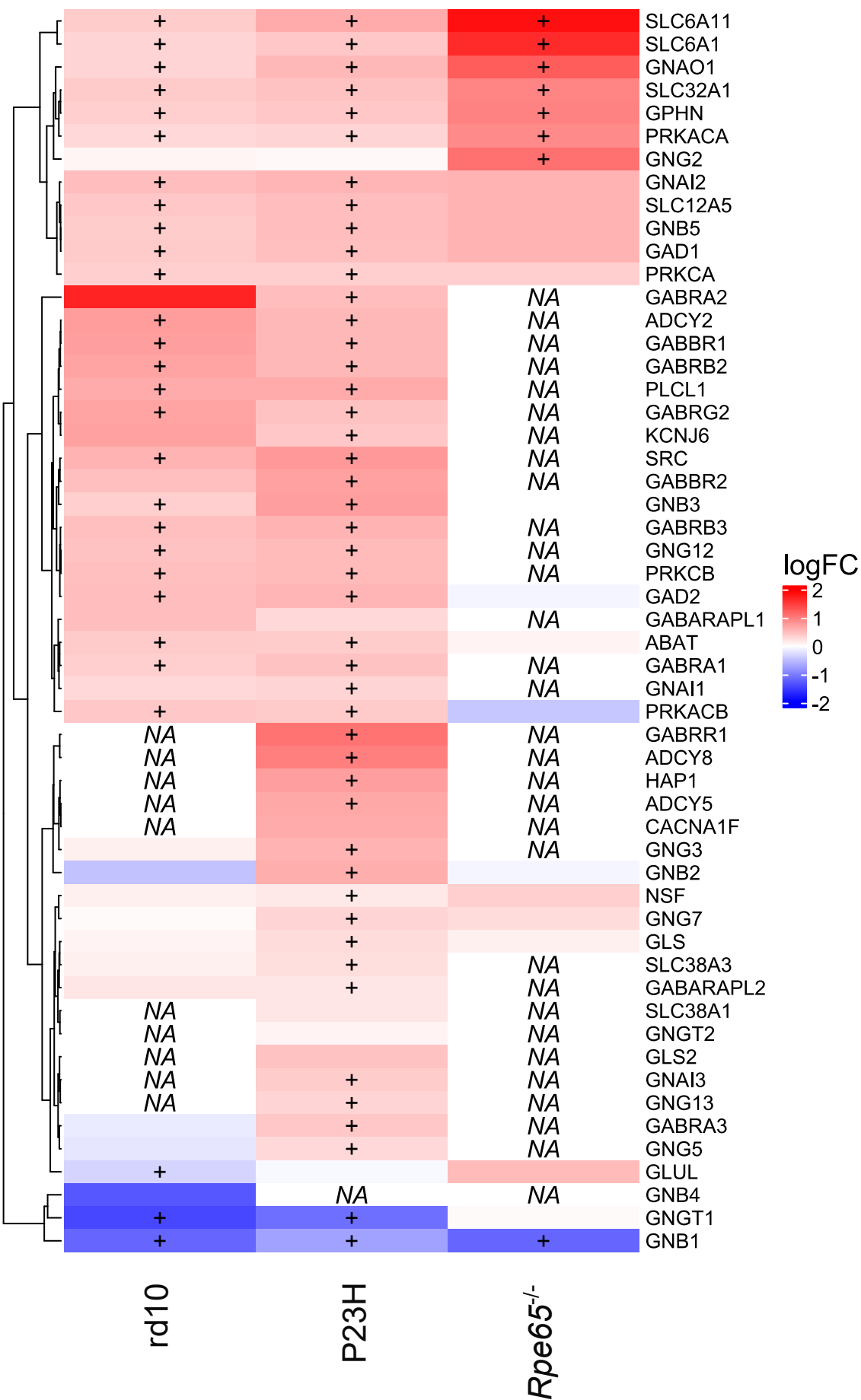

Supplement: Figure S7 [file mmc11.pdf]

# Citrate cycle (TCA cycle)

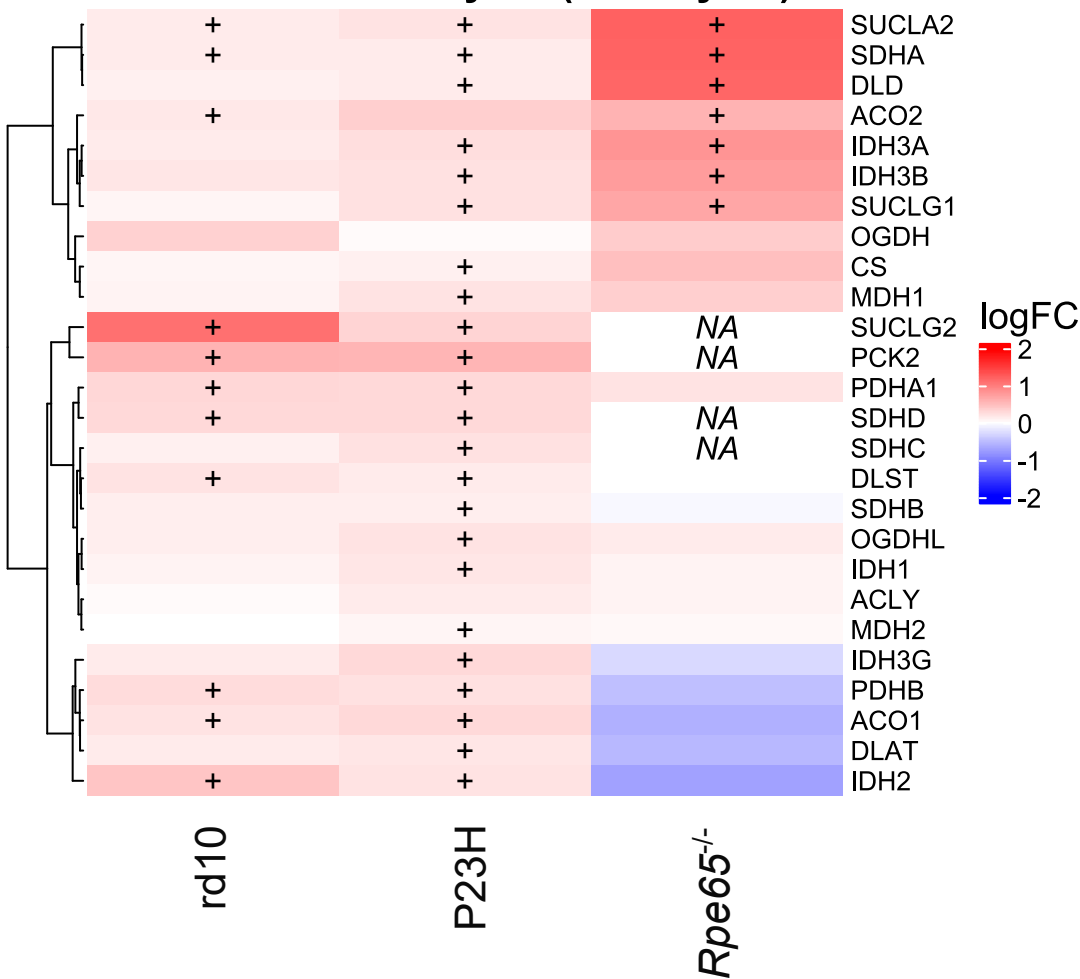

Supplement: Figure S8 [file mmc12.pdf]

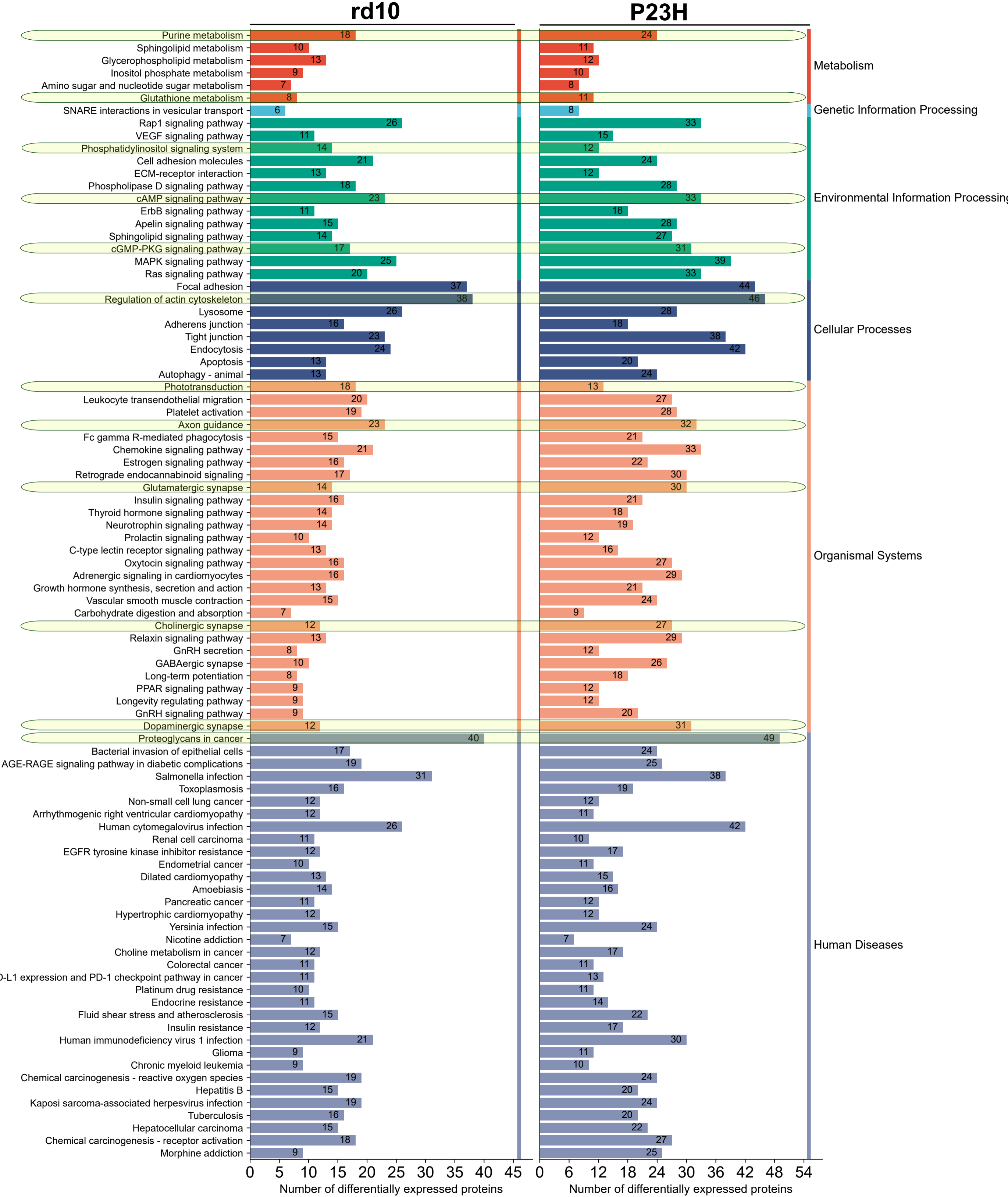

Supplement: Figure S9 [file mmc13.pdf]

Downloaded data and figures may differ in the future.

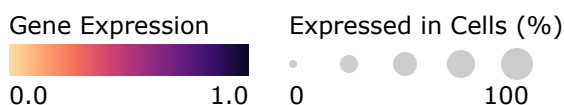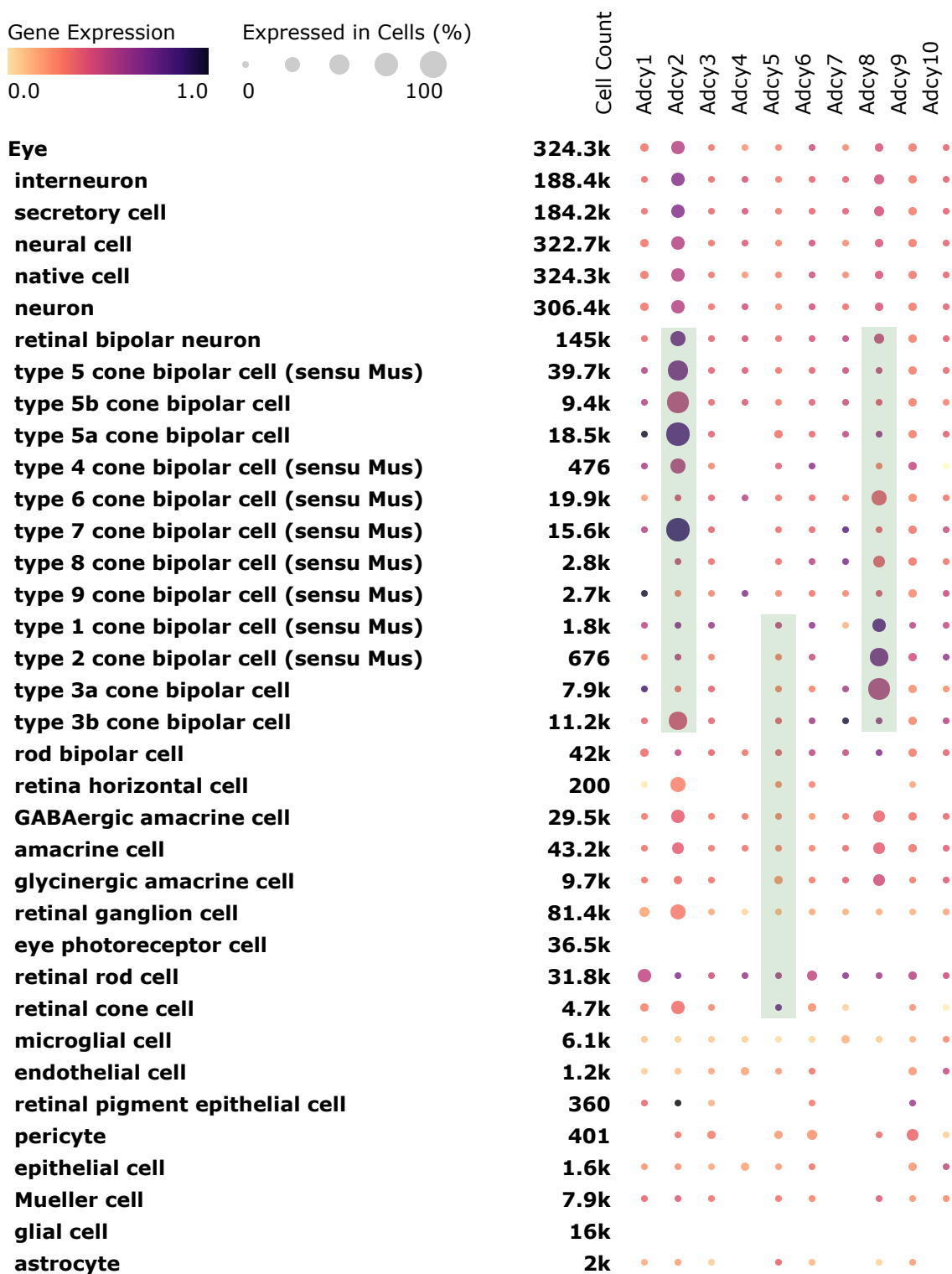

Supplement: Figure S10 [file mmc14.pdf]

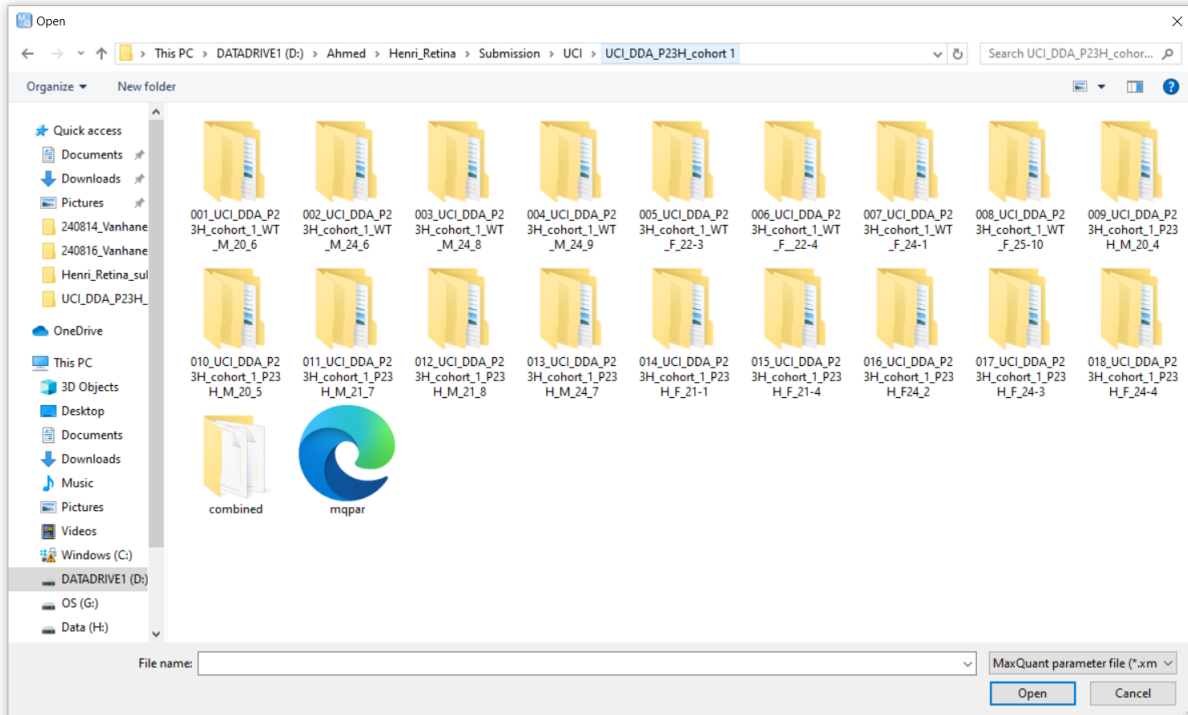

Supplement: Figure S11 [file mmc15.pdf]

# LILQHVQALLVK, Charge 3

File: 20230609\_F1 (RT: 54.37) Reference spectrum

Library: IRD\_UEF\_DIANN\_Skyline\_P23H

Spectrum: 20230609\_F1 (54.37)

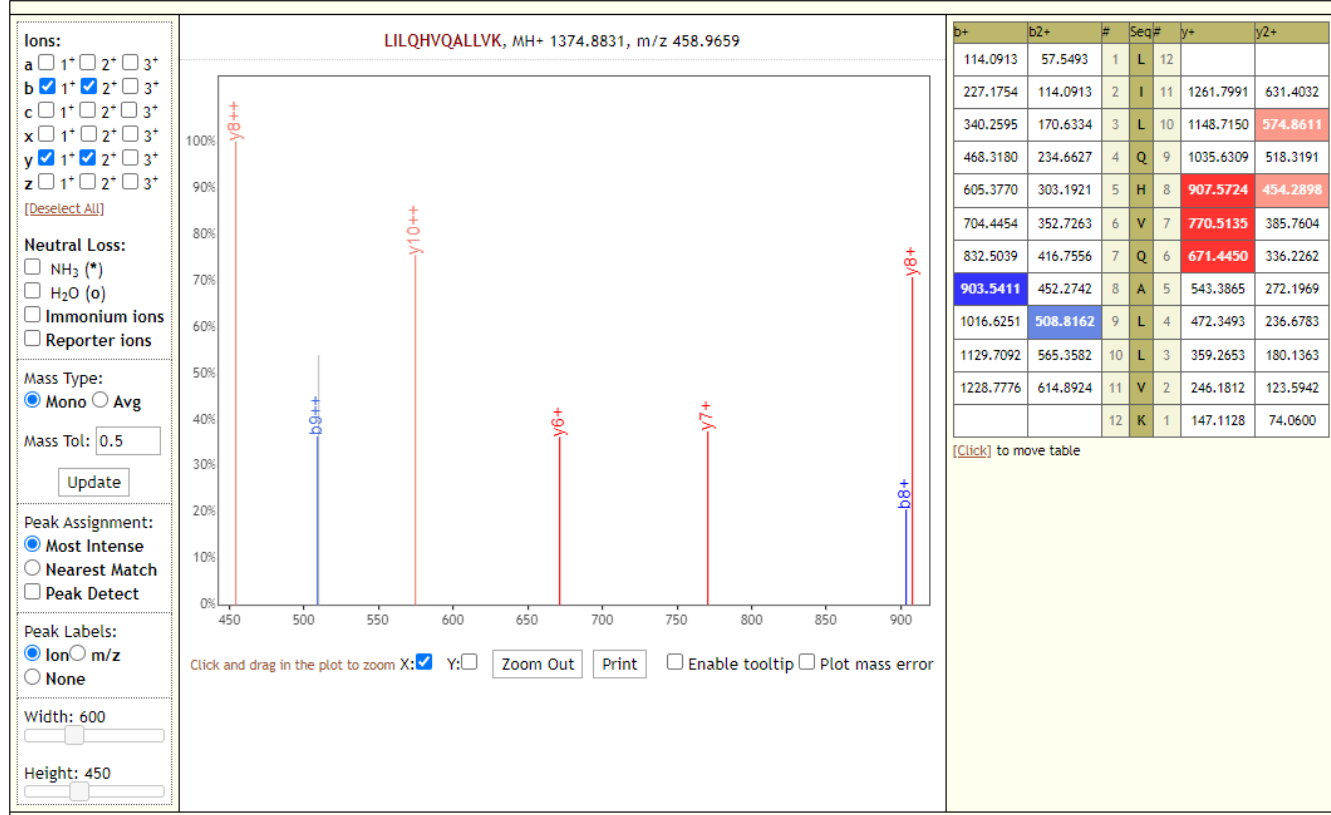

Supplement: Figure S14 [file mmc18.pdf]
