# Supplementary material for: Retinal Proteome Profiling of Inherited Retinal Degeneration Across Three Different Mouse Models Suggests Common Drug Targets in Retinitis Pigmentosa
Source: Mol Cell Proteomics. 2024 Oct 9;23(11):100855. doi: 10.1016/j.mcpro.2024.100855 (PMC11602984; doi:10.1016/j.mcpro.2024.100855)

Sequence	Length	Missed cleavages	Mass	Proteins	Start position	End position	Gene names	Unique (Groups)	Unique (Proteins)	Charges	PEP	Score	id	Protein group IDs	MS/MS Count
ANPYECGFDPTSSAR	15	0	1670.6995	P03899	34	48	Mtnd3	yes	yes	2	3.2023E-14	171.26	2625	1311	29

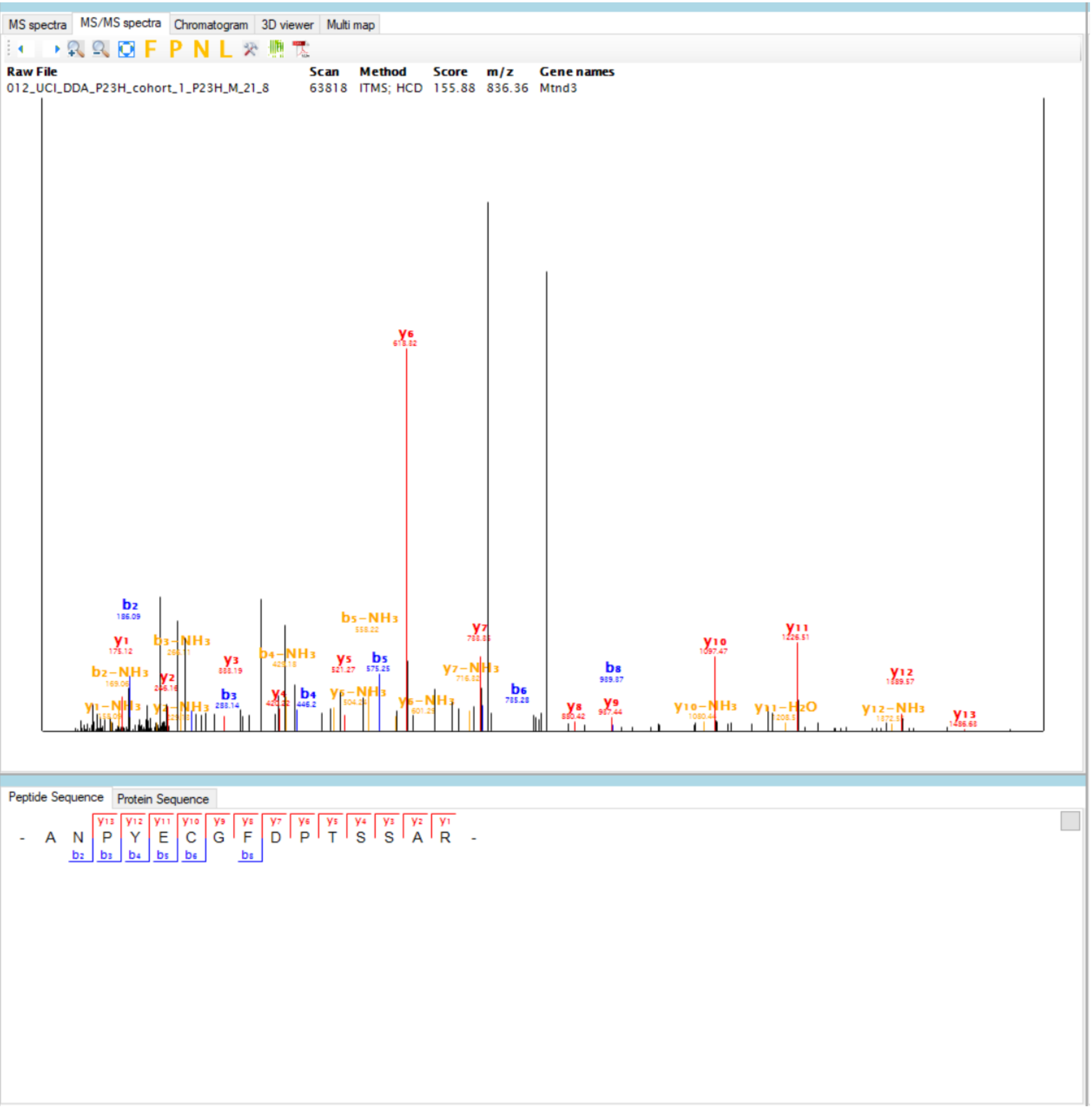

Supplement: Figure S12 [file mmc16.pdf]
