# Supplementary material for: Retinal Proteome Profiling of Inherited Retinal Degeneration Across Three Different Mouse Models Suggests Common Drug Targets in Retinitis Pigmentosa
Source: Mol Cell Proteomics. 2024 Oct 9;23(11):100855. doi: 10.1016/j.mcpro.2024.100855 (PMC11602984; doi:10.1016/j.mcpro.2024.100855)
Supplement: Figure S13 [file mmc17.pdf]

⊟ LILQHVQALLVK, Charge 2

File: 20230609_F5 (RT: 54.33)
Library: IRD_UEF_DIANN_Skyline_P23H
Spectrum: 20230609_F5 (54.33)

Ions:
a ☐ 1+ ☐ 2+ ☐ 3+
b ☑ 1+ ☐ 2+ ☐ 3+
c ☐ 1+ ☐ 2+ ☐ 3+
x ☐ 1+ ☐ 2+ ☐ 3+
y ☑ 1+ ☐ 2+ ☐ 3+
z ☐ 1+ ☐ 2+ ☐ 3+
[Deselect All]

Neutral Loss:
☐ NH_3 (*)
☐ H_2O (o)
☐ Immonium ions
☐ Reporter ions

Mass Type:
◉ Mono ○ Avg

Mass Tol: 0.5

Update

Peak Assignment:
◉ Most Intense
○ Nearest Match
☐ Peak Detect

Peak Labels:
◉ Ion ○ m/z
○ None

Width: 600

Height: 450

LILQHVQALLVK, MH+ 1374.8831, m/z 687.9452

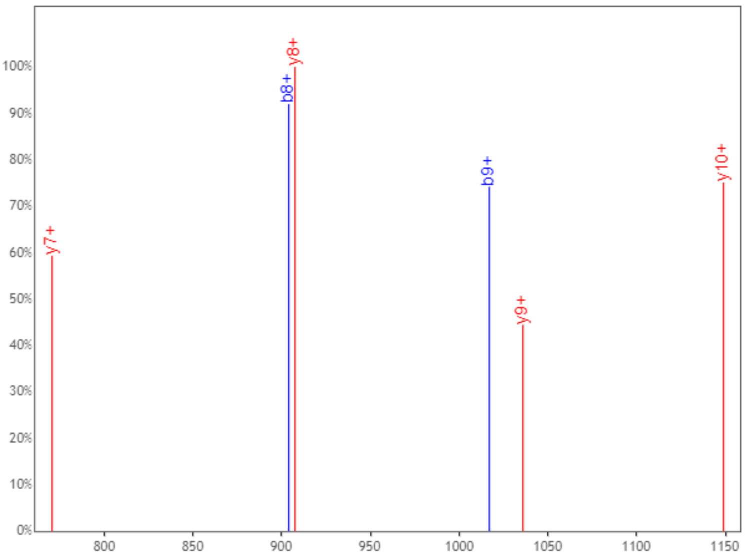

Click and drag in the plot to zoom X: ☑ Y: ☐ Zoom Out Print ☐ Enable tooltip ☐ Plot mass error

b+	#	Seq	#	y+
114.0913	1	L	12	
227.1754	2	I	11	1261.7991
340.2595	3	L	10	1148.7150
468.3180	4	Q	9	1035.6309
605.3770	5	H	8	907.5724
704.4454	6	V	7	770.5135
832.5039	7	Q	6	671.4450
903.5411	8	A	5	543.3865
1016.6251	9	L	4	472.3493
1129.7092	10	L	3	359.2653
1228.7776	11	V	2	246.1812
	12	K	1	147.1128

[Click] to move table
